# The marginal majority effect: When social influence produces lock-in

**DOI:** 10.1126/sciadv.adr4237

**Published:** 2026-02-18

**Authors:** Alexandros Gelastopoulos, Pantelis P. Analytis, Gaël Le Mens, Arnout van de Rijt

**Affiliations:** ^1^ Department of Social and Behavioral Sciences, Toulouse School of Economics, University of Toulouse Capitole, Toulouse 31080, France.; ^2^ Department of Business and Management, University of Southern Denmark, Odense DK-5230, Denmark.; ^3^ Department of Economics and Business, Universitat Pompeu Fabra, Barcelona 08005, Spain.; ^4^ Barcelona School of Economics, Barcelona 08005, Spain.; ^5^ Danish Institute for Advanced Study, University of Southern Denmark, Odense DK-5230, Denmark.; ^6^ UPF-Barcelona School of Management, Barcelona 08008, Spain.; ^7^ Department of Political and Social Sciences, European University Institute, San Domenico di Fiesole (FI) 50014, Italy.

## Abstract

People are influenced by the choices of others, a phenomenon observed across contexts in the social and behavioral sciences. Social influence can lock in an initial popularity advantage of an option over a higher quality alternative. Yet, several experiments designed to enable social influence have found that social systems self-correct rather than lock in. Here, we identify a behavioral phenomenon that makes inferior lock-in possible, which we call the “marginal majority effect”: a discontinuous increase in the choice probability of an option as its popularity exceeds that of a competing option. We demonstrate the existence of a marginal majority effect in several recent experiments and show that lock-in always occurs when the effect is large enough to offset the quality effect on choice but rarely otherwise. Our results reconcile conflicting past empirical evidence and connect a behavioral phenomenon to the possibility of social lock-in.

## Teaser

Popularity trumps quality when people are influenced by popularity ranks rather than counts.

## INTRODUCTION

Whether it is about choosing a party to vote for (*1*), deciding whether to adopt an innovation (*2*), selecting music to listen to (*3*), or deciding whether to get vaccinated during a pandemic (*4*), people tend to select options that many other people have selected before them (*5*–*8*). Work on network externalities, information cascades, and herding in economics (*6*, *9*–*11*); on normative conformism in anthropology and biology (*12*, *13*); and on status-quality dissociation in sociology (*14*) has pointed out that social influence can trigger a reinforcing feedback loop that drives popular options to become even more popular, leading to path dependence and potentially lock-in on inferior outcomes. This hypothesis has found support in historical empirical evidence (*9*, *15*, *16*) and more recently in large-scale experimental studies (*17*, *18*).

Yet, the same hypothesis has also been refuted in experiments across the social and behavioral sciences that were designed to enable social influence. In the classic social conformity paradigm in psychology, social influence cannot sustain most of the responses for the wrong answer (*5*, *19*, *20*). In experiments where the information cascade model predicts that lock-in should occur, majorities favoring the incorrect choice tend to self-correct (*21*). Similarly, experiments on platform choice in economics show that people readily switch to a superior platform when it becomes available, overcoming network effects (*22*). In sociological and political science experiments, social influence fails to popularize mediocre songs, less fit presidential candidates, or bad art (*3*, *20*, *23*). Also, in an experiment on crowd wisdom in the answering of knowledge questions, some questions frequently produced lock-in on the incorrect answer, but other questions never did (*24*). A reconciliation of this mixed body of evidence must state when inferior lock-in occurs and when not.

Previous studies have proposed conditions under which lock-in or herding on inferior options for long stretches of time is more likely to occur. For example, models of information cascades in economics and status-quality dissociation in sociology assert that quality uncertainty increases the probability of settling for an inferior outcome (*11*, *25*, *26*). Studies in the behavioral sciences and complex systems suggest that a higher degree of conformity (i.e., due to the low reliability of asocial information) can increase the probability of herding on inferior options (*27*, *28*). Other authors have focused on the effects of epistemic or behavioral diversity among decision makers, finding that more diversity can reduce the risk of herding on inferior options (*29*) and can help a population adopt a new behavior (*30*). What these studies do not do, however, is reconcile the mixed empirical evidence in terms of a precise condition that is met in cases where inferior lock-in is observed and not when lock-in is not observed.

The contribution of the present paper is to identify such a condition for lock-in and to show that this condition explains the occurrence or absence of lock-in in past experiments. The paper does so by extending a theoretical framework for binary choice under social influence that has appeared in several scientific disciplines in the social sciences (*1*, *6*, *13*, *30*–*32*). The condition we identify relates to the tendency of people to follow the option a majority of others have chosen, regardless of the size of this majority. More precisely, we define the “marginal majority effect” as the increase in the probability that an individual chooses a certain option when this option becomes marginally more popular than its competition—i.e., when the proportion of prior adopters moves from slightly below to slightly above that of a competing option. This sensitivity to marginally higher popularity may reflect a cognitive effect, e.g., if a large number of people use a “follow-the-majority” heuristic (*33*), but it can also be due to other reasons, e.g., when an online algorithm presents options in a way that emphasizes their popularity ranks. We model the marginal majority effect as a discontinuity in the degree of social influence as the support of an option increases, and we show theoretically that when this effect is larger in magnitude than the effect of the quality difference between the two options, then inferior lock-in is always possible (Theorem 2). We also derive a quantitative lower bound for the probability of lock-in as a function of the marginal majority effect and the quality difference (Theorem 3). Thus, although a larger majority can exert larger influence on an individual’s choice, the degree to which people are influenced by marginal majorities affects the occurrence of lock-in on an inferior option.

Majority influence has been one of the main research threads in social psychology following Asch’s seminal work (*5*). Nonetheless, the potential impact of marginal majorities has been largely overlooked as more emphasis was put on the influence of larger majorities. A recent extensive literature review on majority-following rules (*33*), for example, reassesses past theoretical and empirical work on majority-following rules, but without explicit reference to the potential appeal of marginal majorities, as contrasted to larger majorities. In our review of the literature, we were able to identify only a handful of studies that explicitly discuss the potential of marginal majorities to influence decisions (*18*, *34*–*36*). The idea of a discontinuity in the degree of social influence also runs counter to models of majority influence in the social and behavioral sciences, which assume that increasing group or majority size has a gradually increasing impact on the degree of influence (*7*, *12*, *37*–*39*). As a result, the theoretical implications of marginal majorities for lock-in have also not been explored.

After presenting our theory, we provide evidence for the marginal majority effect and its consequences for lock-in by reanalyzing data from three multiple-world experiments on binary choice from diverse contexts (political preferences, matters of fact, and matters of taste). We find that a marginal majority can substantially influence people’s choices, even when this marginal majority is not emphasized in the experimental design. We then show that when the marginal majority effect is larger than the effect of the quality difference between the two items, the social system will exhibit lock-in with probability consistent with our theory. Moreover, our empirical analysis shows that in practice, the converse is also true, i.e., when the marginal majority effect is either nonexistent or smaller than the quality difference of the two items, the system is generally self-correcting (*20*, *21*). Our results thus suggest that the marginal majority effect (i) is prevalent in a variety of settings and (ii) has explanatory power with respect to the occurrence of lock-in. Last, our empirical analysis also tests claims that follow from the existing theory of social influence for binary choice (*6*).

## RESULTS

### Model

**Fig. 1.**
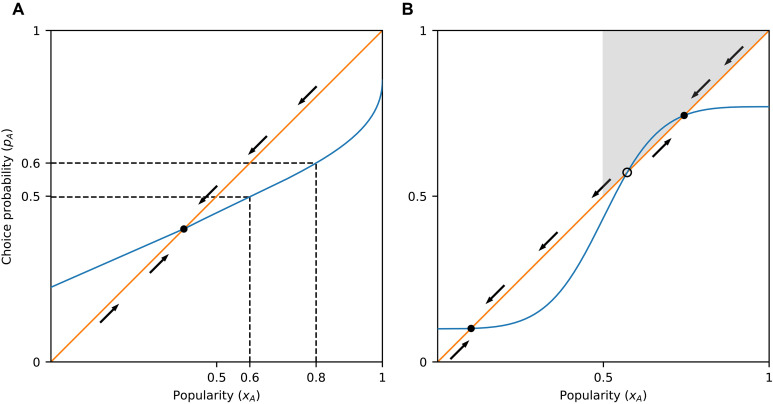
Influence curves and equilibria. (**A**) Probability that an individual chooses item *A* is a function of its current popularity, that is, $p_{A}=f\left(x_{A}\right)$. In the binary choice process described by this influence curve (blue line), if the popularity of item *A* is *x_A_* = 0.8, then it is selected with probability $p_{A}=0.6<x_{A}$, thus driving its popularity downward (indicated with an arrow pointing downward along the diagonal). As the popularity decreases from 0.8 toward 0.6, the choice probability also goes down, driving the popularity further down and so on. A similar argument shows that the popularity is driven upward whenever $p_{A}>x_{A}$. Where the graph intersects the diagonal (orange line), the choice probability is equal to the popularity ($p_{A}=x_{A}$), indicating an equilibrium. (**B**) Graph can intersect the diagonal at multiple points, implying multiple possible equilibria. At the points where the graph crosses the diagonal from above (filled circles), the equilibria are stable, meaning that deviations from those will eventually be corrected (arrows showing the direction in which popularity moves are converging). Where it crosses from below (open circle), there is an unstable equilibrium (popularity from nearby points moves away from that point). The process is lock-in-prone if and only if there exists a stable equilibrium in which the popularity of A is larger than 0.5, i.e., on the right half of the graph. This is equivalent to the curve entering the region y>x>0.5 (shaded region), known as the lock-in region (*20*).

A large number of individuals are presented one after the other with a choice between two options, *A* and *B*. The probability $p_{A}$ that an individual chooses option *A* is an increasing function of the current popularity of *A*, defined as the proportion of previous individuals that have chosen this option, and it is independent of previous individuals’ choices otherwise. That is, there is some nondecreasing function *f* such that $p_{A}=f\left(x_{A}\right)$, where $x_{A}=\frac{n_{A}}{n_{A}+n_{B}}$ is the current popularity of *A* (Fig. 1A). We call this a binary choice process and *f* the influence curve. By convention, we assume that *A* is the inherently less appealing option. This is defined to mean that if the two options were equally popular, then *A* would be no more likely than *B* to be chosen, that is, f(0.5)≤0.5.

Our model does not assume that individuals are homogeneous with respect to preferences or in how they are influenced by others. Instead, $f\left(x_{A}\right)$ represents the probability that a randomly chosen individual chooses option *A*, given *A*’s current popularity. It can be thought of as an aggregation of individual-level influence curves $f_{i}\left(x_{A}\right)$, which can take any form, but we are not modeling them explicitly (see section S1). In the special case that the $f_{i}\left(x_{A}\right)$’s are step functions that take only the values 0 and 1, this is a threshold model with heterogeneous thresholds (*30*). Under the random sampling assumption, the aggregate influence curve, obtained through averaging the individuals level curves, contains all information about the behavior of the system.

### Background and definitions

Because of the inherent stochasticity in individuals’ choices, the popularity of the two options will initially fluctuate randomly. In particular, even if *A* is less appealing than *B*, it is possible that *A* will gain an initial popularity advantage, which will make it more likely to be chosen by subsequent individuals. We focus on whether it is possible for such a popularity advantage to persist in the long term (*6*, *20*).

Under mild technical assumptions (it is enough to assume that *f* has at most finitely many discontinuities), Hill *et al.* (*40*) show that $x_{A}$ eventually necessarily converges to some equilibrium value [Corollary 2.1 in (*40*)]. However, the limit value to which it converges is ex-ante random. Let us denote this (in principle random) limit by $x_{\infty }$. We have the following definition.

Definition 1. A binary choice process is self-correcting if the probability that a majority of individuals chooses the inherently less appealing option in the long term is 0, i.e., $\mathbb{P}\left(x_{\infty }>0.5\right)=0$. Otherwise, it is lock-in-prone.

Although the term “lock-in” sometimes is used to describe any stable majority, here, we will reserve it for referring to a stable majority of the inherently less appealing option $\left(x_{\infty }>0.5\right)$.

The graph of the influence curve *f* can tell us whether the process is lock-in-prone or self-correcting. To avoid technicalities in the statements of the results, we will assume throughout that *f* is nondecreasing, 0<f(x)<1 for all *x*, *f* crosses the diagonal line y=x whenever it meets it, and *f* does not cross the diagonal infinitely many times. The following theorem formalizes the lock-in condition in (*20*). Related results appear in an earlier paper by Arthur (*6*). The proofs of all our mathematical results are given in Materials and Methods.

Theorem 1. A binary choice process with influence curve *f* is lock-in-prone if and only if the graph of *f* enters the region y>x>0.5.

The region y>x>0.5, highlighted in Fig. 1, is the lock-in region defined in (*20*). Figure 1A also gives some intuition about why the theorem holds. A special role is played by the points where the influence curve downcrosses the diagonal line y=x, i.e., points $x_{0}$ for which f(x)>x to the left of $x_{0}$ and f(x)<x to the right. These points are called stable equilibria of *f*, and they are the possible long-term limits for *A*’s popularity (*6*, *40*). We note that analogous properties are well-known for deterministic dynamical systems, and in the context of social influence, they have been used in the study of threshold-based models (*30*, *32*). The model we use here is stochastic is nature. In particular, unlike the threshold model or other deterministic systems, the exact same initial conditions can lead to very different long-term outcomes. The theory developed in (*40*) guarantees that some of the same arguments used for deterministic systems are also valid in our case.

Because *A* is by assumption the less appealing option [f(0.5)≤0.5], it is always possible for option *B* to be (equally or) more popular in the long term; hence, there is always a stable equilibrium $x_{0}\leq 0.5$. If the influence curve enters the lock-in region (i.e., the process is lock-in-prone), then there is another stable equilibrium above *x* = 0.5, implying that there are at least two possibilities for the long-term limit, making the process unpredictable (*6*).

A basic model assumption is that the choice probability depends only on the proportion of individuals that have previously chosen *A* or *B*, no matter how many individuals have come before. This assumption can be relaxed, and *f* can be thought of as describing only the behavior of individuals that come late in the sequence, with Theorem 1 and all statements about the asymptotic behavior continuing to hold (*41*).

Although Theorem 1 gives a powerful characterization of lock-in-prone processes, it does not specify a condition for lock-in in terms of elementary micro-level properties. To evaluate lock-in proneness using Theorem 1, one needs to identify the precise shape of the influence curve and determine whether it enters the lock-in region at any point. In what follows, Theorem 1 will serve as our starting point for identifying two parameters interpretable at the micro-level that together form a precise condition for lock-in proneness. Empirically testing whether this condition holds requires data only on a small region of the influence curve’s domain.

### Main argument: Step-like majority influence and lock-in

**Fig. 2.**
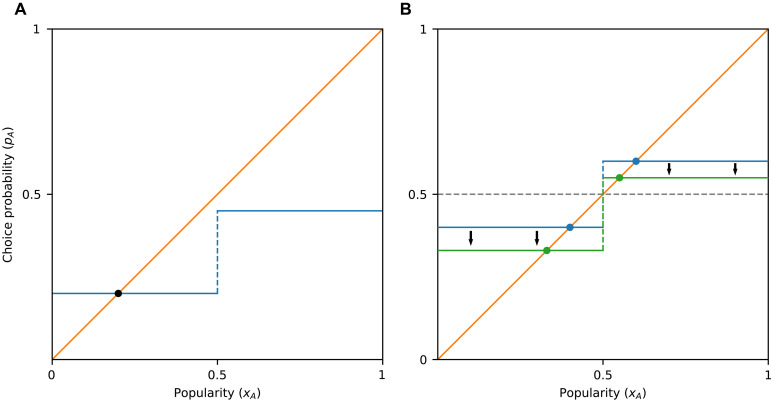
Influence curves and equilibria for majority-based social influence. (**A**) When individuals are influenced only by which option is the majority choice, the graph of *f* consists of two flat parts, on either side of *x* = 0.5. (**B**) If items *A* and *B* are of identical inherent appeal, then the graph is point-symmetric with respect to the point (x,y)=(0.5,0.5) (blue line), with the graph entering the lock-in region immediately to the right of *x* = 0.5. Substituting item *A* with one of slightly smaller inherent appeal results in a downward shift of the two flat parts of the graph (green line). As long as the difference in inherent appeal is small, the departure from a symmetric graph will be small; hence, the influence curve will still enter the lock-in region and exhibit an equilibrium for x>0.5.

To introduce the main idea, let us suppose for a moment that individuals do not take into account the exact proportion of previous individuals who have chosen alternatives *A* or *B*, but instead that they are informed only (or pay attention only to) whether *A* or *B* is currently more popular. Given that an individual does not distinguish between *A* having popularity, e.g., 51 or 95%, their choice probability is the same in both cases. The graph of *f* is thus flat in each of the regions x<0.5 and x>0.5 (Fig. 2).

The key observation is that, under this assumption, and for alternatives with a small difference in inherent appeal, the system is necessarily lock-in-prone. This is because a small difference in inherent appeal implies that the average of the two flat parts of the influence curve is close to *y* = 0.5. As a result, because of the discontinuity at *x* = 0.5, the right part of the influence curve is above *y* = 0.5, and it enters the lock-in region immediately to the right of *x* = 0.5 (Fig. 2B). This statement can be made quantitative, and it applies more generally to any influence curve that exhibits a discontinuity at *x* = 0.5, even if it does not consist of two flat segments, as we show below (Theorem 2).

### Combining step-like and continuous popularity-based influence

We now consider a more general setting, where there can be both step-like and continuously increasing influence. This can model situations in which some individuals pay attention to which option is more popular, while others are subject to a gradually increasing influence as the popularity of one of the options increases. It is also possible that the same individual is subject to both types of influence. For example, one might be weakly inclined to align with the majority even if this majority is small and at the same time become more convinced that the majority is correct as the majority grows.

**Fig. 3.**
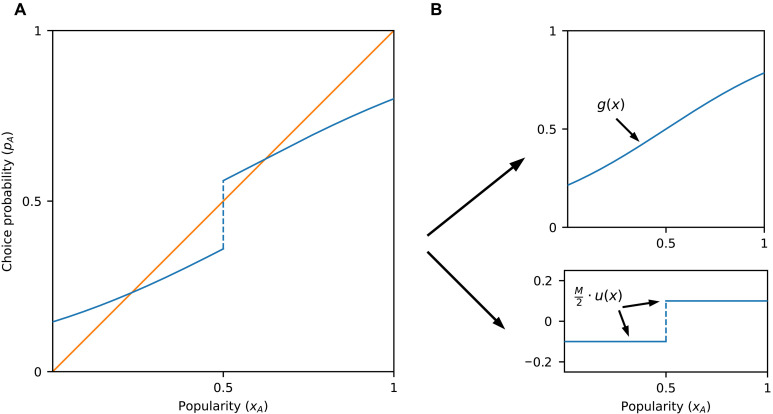
Choice probability versus popularity in the presence of both majority-based and continuously increasing influence. (**A**) Marginal majority effect *M* can be defined as a discontinuity in the influence curve at *x* = 0.5. (**B**) Influence curve *f* with a nonzero marginal majority effect can be written as the sum of two parts, $f\left(x\right)=g\left(x\right)+\frac{M}{2}\cdot u\left(x\right)$, where *g* is continuous and *u* is a step function, with u(x)=±1 if x≷0.5.

In this more general setting, social influence can be modeled through an increasing influence curve with a discontinuity at *x* = 0.5 (Fig. 3A). Such an influence curve can be decomposed into a continuous part and a purely majority-dependent part by writing

$$f\left(x\right)=g\left(x\right)+\frac{M}{2}\cdot u\left(x\right)$$ (1)

where *g* is continuous (at least at *x* = 0.5), *M* is the size of the jump at *x* = 0.5, and *u*(*x*) is a step function that takes the value 1 or −1, depending on whether x>0.5 or x<0.5 (and *u*(0.5) = 0) (Fig. 3B). We call *M* the marginal majority effect.

As in the case of purely step-like majority influence, if the jump that occurs at *x* = 0.5 is large enough to overcome the difference in inherent appeal between the two options, then the graph of *f* will enter the lock-in region immediately to the right of *x* = 0.5, making the system necessarily lock-in-prone (Fig. 3A). To make this precise, we define the difference in inherent appeal *d* as the difference in choice probability of *B* versus *A* when the two items are equally popular, that is, when *x_A_* = 0.5. Note that when *x_A_* = 0.5, the choice probability of *A* is *g*(0.5) and that of *B* is 1−g(0.5); hence, d=(1−g(0.5))−g(0.5)=1−2⋅g(0.5).

Theorem 2. If M>d, then the system is lock-in-prone.

This theorem provides only a sufficient condition for lock-in proneness. However, this condition correctly categorizes most cases of lock-in in several existing social influence experiments (see section “Empirical evaluation of the proposed theory (part 1): Marginal majority effects and inherent appeal differences explain lock-in-proneness”). In other words, for the experiments analyzed below, the condition M>d turns out to be not only sufficient but also approximately necessary.

Compared to Theorem 1, which is an if-and-only-if statement, checking the condition M>d requires much less data because it requires knowing the values of the influence curve only near *x* = 0.5, while Theorem 1 requires knowing the influence curve for all x>0.5. Theorem 2 thus provides a more parsimonious way to assess whether a system can exhibit lock-in.

Although *M* and *d* can individually vary in the interval [0,1], they must always satisfy the inequality M+d≤1. Intuitively, the reason that they cannot both be large is the following: A large discontinuity at *x* = 0.5 (i.e., a large *M*) implies that for popularities just above *x* = 0.5, the inferior option is chosen relatively often (with choice probability at least *M*). This sets a bound on the difference in inherent appeal *d*. More formally, because f(x)≥0 must hold for all *x*, particularly near *x* = 0.5, and *g* is continuous, we get that g(0.5)−M/2≥0 or equivalently g(0.5)≥M/2. Recalling that d=1−2⋅g(0.5), we get d≤1−M⇔M+d≤1.

A particular consequence of the inequality M+d≤1 is that if d>0.5, then we necessarily have M<0.5; hence, M<d, i.e., for differences in inherent appeal that exceed 0.5, the condition of Theorem 2 cannot be satisfied. On the other hand, if M>0.5, we get similarly that M>d and Theorem 2 applies. Therefore,

Corollary 1. If M>0.5, then the system is lock-in prone.

This corollary gives a criterion for lock-in proneness that is independent of the difference in inherent appeal of the two items. However, marginal majority effects this large might be unrealistic in most situations, and in the datasets, we analyze this criterion is never satisfied.

### Probability of lock-in

While Theorem 2 gives a sufficient condition for a binary choice process to be lock-in-prone, it does not say anything about the likelihood of lock-in. This is the focus of the following theorem.

Theorem 3. If M>d, then the probability of lock-in $p_{L}≔\mathbb{P}\left(x_{\infty }>0.5\right)$ satisfies

$$p_{L}\geq \frac{2\left(M-d\right)\left(1-d\right)}{\left(1-d\right)\left(1-d+M\right)+2M}$$ (2)

**Fig. 4.**
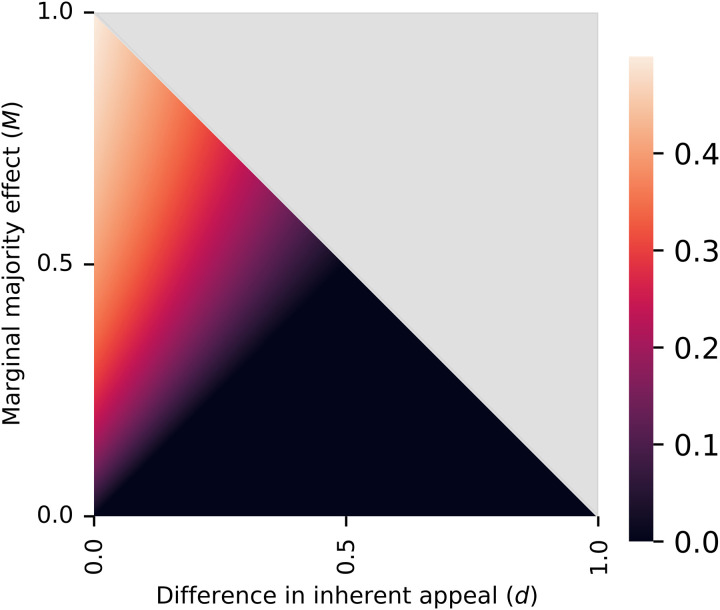
Lower bound for lock-in probability ℙ (*x*_∞_ > 0.5) as a function of *M* and *d*. For each pair of *M* and *d* satisfying M>d, a lower bound for the lock-in probability is given by Eq. 2. When M<d, there is no guarantee of lock-in proneness, i.e., the lock-in probability can be as low as 0 (black region). The gray region corresponds to impossible parameter combinations.

Theorem 3 gives a lower bound for the probability of lock-in when the condition M>d is satisfied (see also Fig. 4). For example, if *M* = 0.2 and *d* = 0.1, then we get $p_{L}\geq \frac{2\cdot \left(0.2-0.1\right)\cdot \left(1-0.1\right)}{\left(1-0.1\right)\cdot \left(1-0.1+0.2\right)+2\cdot 0.2}=\frac{0.18}{1.39}\approx 13\%$. Such a prediction can be tested in experiments with multiple independent trials (see section “Empirical evaluation of the proposed theory (part 2): Frequency of lock-in”). In the Supplementary Materials, we further derive an expression for the exact probability of lock-in in the case where the influence curve is a step function (theorem S1).

### Continuous influence curves

Theorem 2 implies that a discontinuity in the influence curve is sufficient for lock-in proneness, as long as the difference in inherent appeal of the two options is small. In contrast, a process with a continuous influence curve does not have to be lock-in-prone, even if the difference in inherent appeal is small. In Fig. 1, for example, even if the value of *f* was very close to the diagonal at *x* = 0.5, which would imply a tiny difference in inherent appeal, *f* would not enter the lock-in region.

**Fig. 5.**
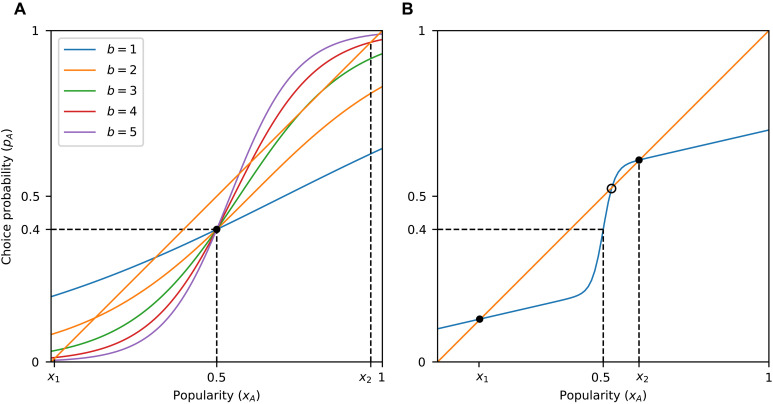
Continuous influence curves and lock-in proneness. (**A**) Logistic influence curve (Eq. 3) with *d* = 0.2 and varying *b*. For b<3, the process is self-correcting, while for b>3, it is lock-in-prone with stable equilibria at $x_{1}\approx 0$ and $x_{2}\approx 1$ (here shown for the case *b* = 4). (**B**) Given that *A* is an inherently less appealing option [f(0.5) is below the diagonal], between *x* = 0.5 and any downcrossing on the right half of the line ($x_{2}$) there is an upcrossing. As a result, for a downcrossing to occur above but close to *x* = 0.5, *f* must have a very steep slope near *x* = 0.5, which quickly diminishes shortly, after, i.e., it must exhibit a marginal majority-like effect.

This is not to say that a binary choice process with a continuous influence curve cannot be lock-in-prone. Figure 5A shows the graph of the logistic function

$$f\left(x\right)=\frac{1}{1+\frac{1+d}{1-d}\cdot e^{b\left(1-2x\right)}}$$ (3)

a standard function used to model binary choice in the social, behavioral, and management sciences, which has been often used to describe forms of social influence (*7*, *24*, *39*, *42*–*44*). Here, *d* is the difference in inherent appeal as defined earlier (in the graph of Fig. 5A; *d* = 0.2), while *b* controls the slope. For small values of *b*, the graph does not enter the lock-in region, making the system self-correcting, but for larger values (*b* > 3 when *d* = 0.2), the graph does enter the lock-in region, making the system lock-in-prone. In the latter case, however, the two stable equilibria of the system lie very close to *x* = 0 and to *x* = 1, respectively, which correspond to winner-take-all situations. This is the case because these large values of *b* imply very strong social influence.

More precisely, the logistic equation has the property that social influence is relatively much stronger when $x_{}$ approaches extreme values (0 or 1) compared to when it takes more moderate values. This is common in systems that exhibit network externalities (*45*). When this is the case, equilibria are likely to occur only at values x<0.5 or well above *x* = 0.5, as suggested by Fig. 5A. In contrast, when social influence is highly sensitive to differences in popularity only near *x* = 0.5 (e.g., in the presence of marginal majority effects in otherwise relatively flat influence curves), equilibria just above *x* = 0.5 are possible (Figs. 2B, 3A, and 5B).

### Empirical evidence

We empirically evaluate our theoretical results against the findings of experiments on binary choice under social influence. We analyze data from three recent multiple-world experiments that have reported differential findings regarding the possibility of lock-in, namely, the ones reported in (*20*) (no lock-in), (*18*) (lock-in commonly observed), and (*24*) (lock-in observed only in some of the experimental items), to which we refer below as V2019, MDRT2019, and FV2021, respectively (Table 1). These experiments are compatible with our theoretical framework because they involve choice between two alternatives only, participants choose in a sequence, and each participant has aggregate information about the choices of all past participants in the same trial. In addition, these experiments are suitable for assessing lock-in proneness because they either involve numerous trials per experimental item or include trials where the inherently less attractive option is given an initial popularity advantage.

**Table 1.** Multiple-world experiments reanalyzed in this study. The last three columns show the number of items (questions), number of worlds (trials) per item, and number of participants per world in each experiment. V2019 had one world with 530 participants and one with 3500 participants per item. FV2021 had 10 to 15 worlds of 100 participants and 20 to 30 worlds of 15 participants for each item.

Dataset	Publication	# Items	# Trials	# Participants
V2019	(*20*)	7	2	530/3500
MDRT2019	(*18*)	20	8	230
FV2021	(*24*)	30	30–45	15/100

In the analysis below, we first estimate the influence curves and check whether marginal majority effects are present. We then evaluate whether the empirical occurrence of lock-in and the long-term popularities are consistent with existing theory [particularly, Theorem 1 and (*6*, *20*)]. Although this analysis concerns prior theoretical work, it complements our main argument by providing empirical support for the framework that we build upon. Next, we empirically evaluate the theory developed here, specifically whether the values of *M* and *d* explain lock-in proneness (Theorem 2) and the frequency of lock-in (Theorem 3). Last, we compare our theory to a simpler model that asserts that only the difference in inherent appeal *d* matters. The analysis reported in the main text uses all the data available both for estimating influence curves/marginal majority effects and for examining the occurrence of lock-in; hence, it examines the explanatory power of the theory but not its out-of-sample predictive power (*46*). In the Supplementary Materials (section S5), we report an ancillary analysis that uses separate subsets of the data for estimating the influence curves/marginal majority effects and for testing the occurrence of lock-in. This out-of-sample analysis is limited by the available data, but the results are largely consistent with those reported here.

### Datasets

#### *Dataset 1: V2019* (*20*)

**Fig. 6.**
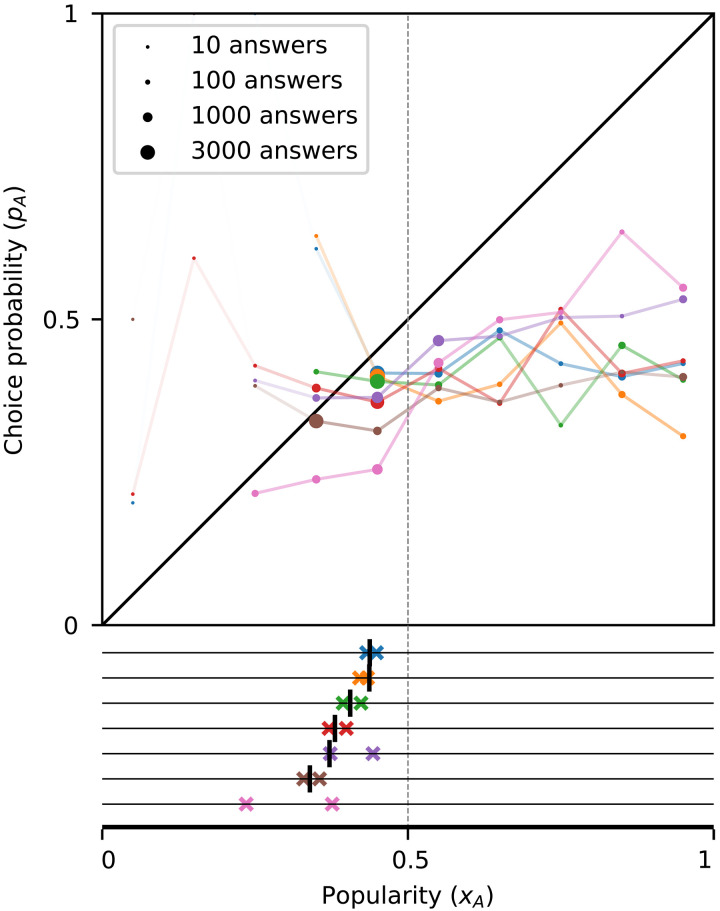
Influence curves and end-of-trial popularities for V2019. (**Top**) For each question, influence curves are estimated by grouping answers from participants in all trials into 10 intervals based on the popularity of option *A* at the time (see Materials and Methods). The area of each circle is proportional to the number of answers available for the estimate. For better readability, line segments whose one (or both) end points are based on a small number of answers are made more transparent. Given that none of the influence curves enters the lock-in region, the theory asserts that lock-in is impossible in all cases (Theorem 1). (**Bottom**) Colored crosses indicate the end-of-trial popularities $x_{\infty }$ of option *A* in the two trials of the experiment (one horizontal line per question). In all cases, $x_{\infty }<0.5$. The points where the influence curves downcross the diagonal are indicated with vertical bars, and questions are ordered by this value. These points are the theoretically projected long-term proportions of *A*, and they agree reasonably with the observed end-of-trial proportions. Note that in one of the two trials for each question, option *A* was given a large artificial advantage in popularity; hence, the lack of lock-in is strongly indicative of its impossibility.

This experiment included seven binary choice questions that were primarily on matters of taste and had no correct answer (e.g., choose the piece of art that you like more). Participants were shown the number of previous participants that had chosen each of the two answers. There were two independent worlds: In the first world, there were ~530 participants who were informed of the true number of previous answers; in the second world, there were 3500 participants, but the less attractive option (identified in a prestudy) was given an artificial initial advantage of about 110 to 10 in terms of previous answers. The true counts of answers during the experiment were added to this artificial initial count. The influence curves for the seven questions are shown in Fig. 6.

#### *Dataset 2: MDRT2019* (*18*)

Participants in the United States reported their political orientation (democrat or republican) and then answered whether they agreed or not with a series of 20 statements on social issues. For each statement, the participant was informed whether the proportion of participants supporting the statement was larger among republicans or democrats in their world (in eight independent worlds + two control worlds), but not by how much. This was communicated both in a text message on the screen and by the font color of this message (red or blue). Thus, in this experiment, a marginal majority must have the same effect as a much larger majority by design because the information provided to the participants in both cases was the same.

**Fig. 7.**
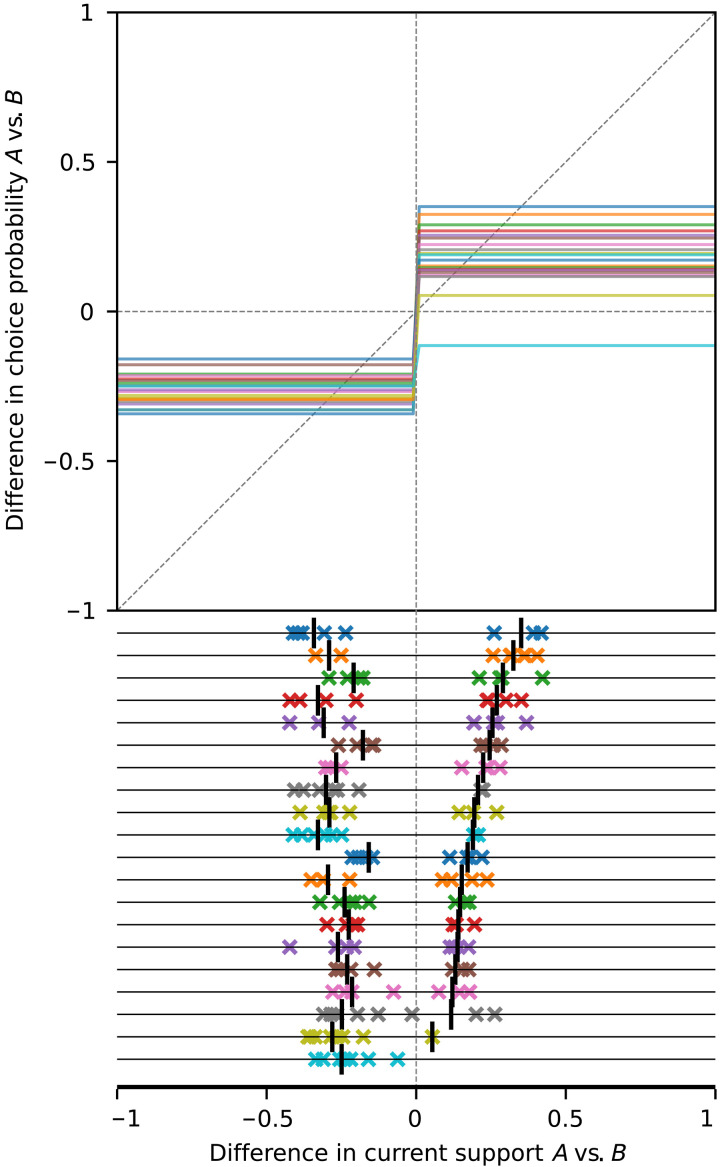
Influence curves and end-of-trial popularities for MDRT2019. Participants were informed whether the support for a statement so far was larger among democrats or republicans; hence, the variables of interest are the difference in the percentage support so far between the two parties and the difference in choice probability between the two parties, both in the range [−1,1]. For each statement, party *A* is defined as the party that is inherently less likely to support the statement. The lock-in proneness condition is that the influence curve enter the region y>x>0. Because participants did not know the exact difference in current support, but only whether it was positive or negative, we force the influence curves to be flat on either side of *x* = 0 and make a single estimate on each side (see Materials and Methods). In all but one case, the influence curve enters the lock-in region (equivalently, there are downcrossings on the right half of the graph, indicated by vertical bars on the bottom panel); thus, the theory asserts that lock-in is possible. In all but the same one case, end-of-trial majority support by party *A* ($x_{\infty }>0$) is observed in at least one of the trials (colored crosses on the right half of the graph), which suggests lock-in. Items are ordered by descending value of the rightmost downcrossing (vertical bar).

Furthermore, because the information provided involved the comparison of two proportions (one for democrats and one for republicans), a slight modification to our framework is needed to apply it to this case. Specifically, a larger republican (democrat) support can be sustained if, given such a larger republican (democrat) support, the probability of agreeing with the statement is also larger for republicans (democrats). The variables of interest here are the difference in the two proportions ($x=x_{A}-x_{B}$) and the difference in the choice probabilities ($y=p_{A}-p_{B}$) for the two groups, both taking values in [−1,1] (Fig. 7). With the convention that *A* denotes the party that is inherently less likely to support a statement, the lock-in region is now the region y>x>0.

#### *Dataset 3: FV2021* (*24*)

**Fig. 8.**
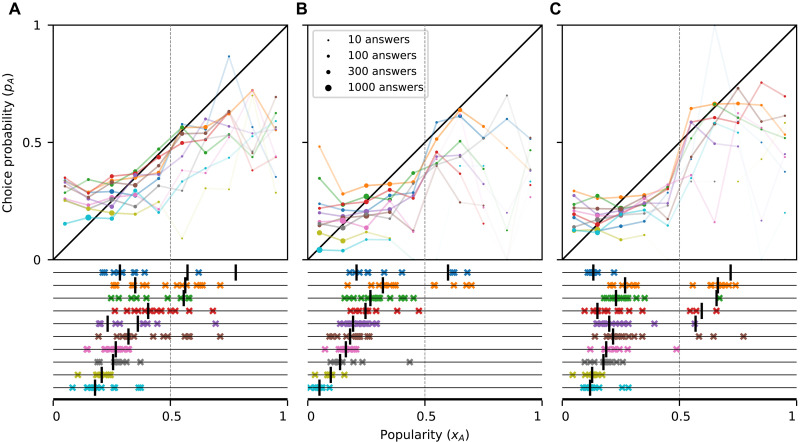
Influence curves and end-of-trial popularities for FV2021. Similar to Fig. 6, but for the FV2021 dataset. In this experiment, one of the two possible answers for each question was correct, and participants were incentivized to answer correctly. Option *A* is defined as the less popular option in the control condition, independently of being the correct one or not. There were 10 visual judgment questions (**A**), 10 art history questions (**B**), and 10 geometry questions (**C**). Across panels, the lock-in region is entered in nine cases (downcrossings on the right half of the graph, indicated in the bottom panel with vertical bars), and in eight of them, end-of-trial majorities of option *A* are observed (in at least one of the trials; colored crosses), which is evidence for lock-in. There are also five cases in which the lock-in region is not entered, but end-of-trial majorities for *A* are still observed (false negatives). Only end-of-trial proportions from longer trials (100 participants) are taken into account and shown in the bottom panels. In each panel, items are ordered by descending value of the rightmost downcrossing (vertical bar).

Participants were given 30 trivia tasks—10 of which were visual puzzles, 10 were art-related questions, and 10 were geometry questions—and they were informed about the number of previous participants in their world that had chosen each of the two possible answers. For each question, there were either 10 or 15 worlds with 100 participants each and either 20 or 30 worlds with 15 participants each. Participants were allowed to abstain. Each question in this study had a definite correct answer. However, for consistency with the other datasets and the theory, we define option *A* (the inherently less appealing option) as the option that fewer people selected in the control condition (no social influence), independently of whether it was the correct answer or not. The influence curves are estimated as before and shown in Fig. 8.

### Evidence for marginal majority effects

We begin with some descriptive statistics regarding the presence and magnitude of marginal majority effects, which we estimate at the level of single experimental items (questions).

The marginal majority effects are most straightforward in MDRT2019 (Fig. 7), where social influence is purely majority-driven. The discontinuities of the influence curves at *x* = 0 in this experiment vary between 0.13 and 0.7.

In V2019 (Fig. 6), all influence curves are relatively flat away from the middle, while for several of the questions, we observe a pronounced increase in the influence curve between the two central bins (changes in the range 0.055 to 0.17 for four of the questions), which remains relatively constant as we decrease the bin size (fig. S8) and is thus indicative of a marginal majority effect. In other words, most of the social influence observed is a result of a marginal majority or marginal majority-like effect. Note that in this experiment, the majority choice was not made visually salient on the experiment’s screen (the positions of the options were fixed), but it was instead inferred by the participants from the number of choices indicated under the items. Despite the existence of a marginal majority effect in several cases, this was not large enough for the influence curve to enter the lock-in region in any of the questions.

In FV2021, there is also a sharp increase in choice probability as popularity crosses *x* = 0.5, while the choice probability is relatively insensitive to popularity away from *x* = 0.5 (Fig. 8). This effect is especially pronounced for the geometry questions (Fig. 8C) and, to a somewhat lesser extent, for the art questions (Fig. 8B). The size of the change between the two central bins varied substantially across questions, but it exceeded 0.15 in 8 of the 28 questions for which there were data. We note that these estimates are substantially noisier than for the other two datasets due to the limited available data (see fig. S2), but they remain relatively robust as we vary the bin size (fig. S8). In many cases, the increase in the central region of the graph was large enough for the influence curve to enter the lock-in region immediately to the right of *x* = 0.5.

### Empirical support for previous theory

In this section, we comment on how well the empirical evidence supports two claims that follow from previous theory: that a process is lock-in-prone if and only if the influence curve enters the lock-in region [see our Theorem 1 and (*20*)], and that the stable equilibria of the influence curve are the possible long-term popularities (*6*). The analyses are again at the level of single experimental items. In what follows, we will say that “lock-in occurs” if the end-of-trial choice proportions favor the inherently less appealing option (i.e., option *A*) in at least one of the trials.

#### Influence curves and lock-in

In V2019, the influence curves do not enter the lock-in region for any of the seven questions (Fig. 6) and, consistent with the theory, no lock-in occurs. While in this experiment there were only two trials per question, in one of these trials, option *A* was given a large initial advantage; hence, the absence of lock-in is strongly indicative of its impossibility. In MDRT2019, the influence curves enter the lock-in region in 19 of the 20 cases and lock-in occurs in the same 19 questions (0 misclassified items) (Fig. 7). Last, in FV2021, the influence curves enter the lock-in region in nine cases, in eight of which lock-in occurs (Fig. 8). Lock-in also occurs in 5 of the 21 the items for which the influence curve does not enter the lock-in region, for a total of 6 out of 30 misclassified items for this dataset (6 out of 57 for the three datasets together). The results are similar when we use a different number of bins (figs. S3 to S6) and in an out-of-sample prediction analysis (see section 5.2 in the Supplementary Materials).

The misclassifications obtained for some questions can be attributed to several reasons. First, we define lock-in based on the end-of-trial proportions. It is possible that trajectories in which option *A* had an end-of-trial majority would have eventually been corrected had the trial been longer. This is especially likely for questions in which the end-of-trial proportions have high dispersion (see, e.g., items 4 and 6 in Fig. 8A), indicating an absence of convergence within the time horizon of the study.

Another source of error is of course estimation error. For example, in the only question for which lock-in is not observed although the influence curve enters the lock-in region, the relevant empirical estimate is highly unreliable: The unique point that lies in the lock-in region is estimated based on the responses of only *n* = 4 participants (item 1 in Fig. 8C). In addition, for three of the five cases where lock-in is observed but the influence curve does not enter the lock-in region, it misses it by a small margin (item 6 in Fig. 8A, item 2 in Fig. 8B, and item 6 in Fig. 8C; the distance from the lock-in region is in all cases smaller than 0.02; *n* = 296, 318, and 65, respectively). Apart from the inherent noise in estimating the influence curve with a finite amount of data, note that we are also averaging over bins of size 0.1; it is likely that the true influence curve enters the lock-in region in a subset of the bin. Consistent with this observation, we find that the influence curve for each of the abovementioned three items enters the lock-in region for different choices of bin sizes (figs. S4 to S6). However, choosing a smaller bin size reduces the data per bin and thus increases the noise.

All in all, the available data provide substantial support for the theoretical claim that a binary choice process is lock-in-prone if and only if the influence curve enters the lock-in region [Theorem 1 and (*20*)]. An out-of-sample analysis (section 5.2 in the Supplementary Materials) further provides moderate support that entering the lock-in region is even *predictive* of lock-in proneness.

#### Influence curves and end-of-trial proportions

Another important assertion of the theoretical model we have used is that the stable equilibria of the influence curve (points where it downcrosses the diagonal) are the possible long-term limits for the popularity (*6*). This is empirically confirmed visually in Figs. 6 to 8: The majority of the points in the bottom panel are positioned near the stable equilibria suggested by the influence curves in the top panels, again providing substantial support for the explanatory power of the theory. The results are similar in an out-of-sample prediction analysis (figs. S9 and S10), thus providing moderate support also for the predictive power of the result.

### Empirical evaluation of the proposed theory (part 1): Marginal majority effects and inherent appeal differences explain lock-in proneness

**Fig. 9.**
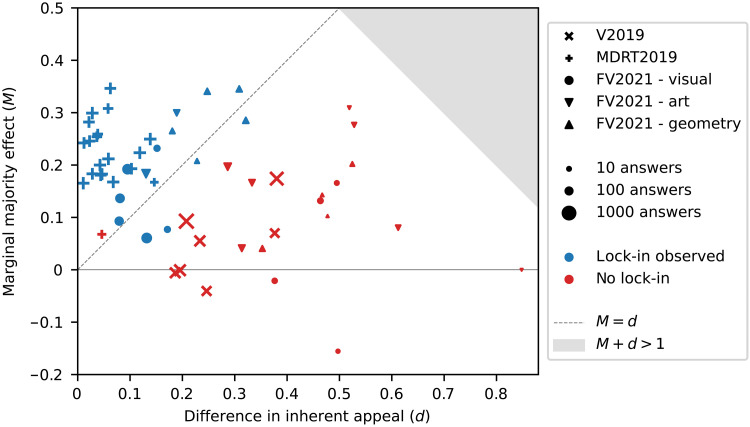
Summary of marginal majority effect versus inherent appeal difference for all datasets. Each data point corresponds to one experimental item (question). The marginal majority effect *M* is estimated as the increase in the influence curve between the two central bins (see Fig. 6), and the difference in inherent appeal *d* is estimated as the difference in choice probability of the two options in the control experiments (no social influence). The definitions of both *M* and *d* are modified suitably in the case of MDRT2019, where they reflect the difference of the corresponding variables for two populations (see Materials and Methods). Blue points correspond to questions in which lock-in was observed, i.e., option *A* had an end-of-trial majority in at least one of the trials, while red color indicates the absence of lock-in. The size of the markers reflects the number of data available for the estimation of *M*. The gray region corresponds to theoretically impossible parameter combinations (M+d>1). The theory asserts that for all points above the dashed gray line (M=d), lock-in is possible, while no claim is made for points below the dashed line. It turns out that for the datasets examined, the condition M>d is highly accurate even as an if-and-only-if condition for the possibility of lock-in.

We now turn to the empirical evaluation of the theoretical results introduced in the present article (Theorems 2 and 3). Figure 9 summarizes the marginal majority effect *M* and inherent quality difference *d* estimates for all items in the three experiments. The distribution of standard errors for these estimates is presented in fig. S2. We note that because in MDRT2019, the influence curve is defined as the difference of two choice probabilities (among supporters of the two parties), the definitions of *M* and *d* are suitably modified in order for Theorem 2 to remain valid. In particular, *M* is equal to half the size of the discontinuity in Fig. 7 (see Materials and Methods).

Figure 9 provides strong empirical support for the claim that the condition identified in Theorem 2, namely, that the marginal majority effect that exceeds the difference in inherent appeal of the two options (M>d) explains lock-in proneness at the level of individual experimental items. Specifically, in almost every case that this condition is satisfied, lock-in is observed (28 of 29 cases or 96.5%), i.e., the inherently less appealing option has an end-of-trial majority in at least one of the trials. Also, although Theorem 2 makes no statement when M≤d, lock-in rarely occurs in this situation (5 of 26 cases or 19%; lock-in also does not occur for two items for which there were no data to estimate *M*). In other words, while Theorem 2 identifies M>d as merely a sufficient condition for lock-in proneness, in practice, it works as both sufficient and necessary, being equally accurate with the true if-and-only-if condition of Theorem 1 despite depending on much less data. The proof of Theorem 2 suggests that the conditions of the two theorems should be roughly equivalent when the most likely place for the influence curve to enter the lock-in region is immediately to the right of *x* = 0.5.

Figure 9 explains the variation in the possibility of lock-in across the three experimental studies: In MDRT2019, the marginal majority effect was substantial in almost all of the cases, and the difference in inherent appeal was generally small, which explains why lock-in was observed in all questions but one. In V2019, the marginal majority effect was nonexistent to moderate, and the difference in inherent appeal was moderate to large. As a result, lock-in was not possible. Last, in FV2021, both the differences in inherent appeal and the marginal majority effects varied in magnitude, leading to lock-in only in a subset of the questions.

In section 5.3 of the Supplementary Materials, we report an out-of-sample analysis, which provides moderate support even for the predictive power of the inequality M>d as a sufficient condition for lock-in proneness.

### Empirical evaluation of the proposed theory (part 2): Frequency of lock-in

In this section, we evaluate how well our theory explains the frequency of lock-in occurrence. Recall that Theorem 3 gives a lower bound for the lock-in probability in a single trial, $p_{L}$, when M>d. This estimate can thus be used to obtain a *p*-value for the observed number of lock-in occurences in a given number of trials. For example, if $p_{L}$ = 0.3, then the probability of not observing any lock-in in 10 trials is ${\left(1-0.3\right)}^{10}\approx 0.028$.

We find that for any individual item, the lower bound obtained by Eq. 2 does not exclude the statistical possibility at a 0.05 confidence level that no lock-in occurs in the number of trials used in these experiments, i.e., the rejection region is empty (smallest *p*-value for 0 lock-in occurrences across items in the three experiments is 0.066). Thus, the available data does not allow to test Theorem 3 at the level of single experimental items. However, at the aggregate level, the values obtained by Eq. 2 imply that lock-in must occur in at least 32 of the 280 total trials for the 29 items that satisfy the condition M>d (95% one-sided confidence interval; see the Materials and Methods). In other words, the rejection region is 0 to 31 lock-in occurrences. The actual number of lock-in occurrences in these trials is 103, which is consistent with the theoretical expectation. We note that while the test might appear to have low power, given the large nonrejection region (any number between 32 and 280), the baseline frequency of lock-in is generally small. For example, in a total of 249 trials for 26 items for which M>d is not satisfied, lock-in occurs only eight times. Thus, the test provides moderate support for the explanatory power of Theorem 3 for the frequency of lock-in. In section S2 of the Supplementary Materials, we derive and test a more precise estimate of the lock-in probability for the MDRT2019 dataset (theorem S1), providing further support for the theory. Last, an out-of-sample test provides moderate support also for the predictive validity of Theorem 3 (section S5.4 in the Supplementary Materials).

### Comparison with a simpler model

A simpler model that one could put forward for explaining the possibility of lock-in is one that takes into account only the difference in inherent appeal *d*. Such a model would state that for items that are of similar inherent appeal (*d* small), the binary choice process is lock-in-prone, but if they differ by a lot in terms of inherent appeal (*d* large), the process is self-correcting.

Figure 9 illustrates that while this simpler model would successfully explain the possibility of lock-in for extreme values of *d*, it would be insufficient in an intermediate range of values (0.2<d<0.35), where the criterion M>d or alternatively the difference *M-d* performs much better. To formally compare the explanatory power of the value of *d* versus that of *M-d* for lock-in proneness, we performed binary regression (on the entire dataset) with the occurrence of lock-in (lock-in proneness) as the binary-response variable and *d* in one case and *M-d* in the other as regressors. We applied both a parametric method (logistic regression) and a nonparametric one (Nadaraya-Watson kernel regression). In the case of logistic regression, we introduced an intercept when using *d* as a regressor, but not when using *M-d*. The reason is that our theory identifies the value M−d=0 as a threshold, while in the case of *d*, there is no ex-ante prediction regarding the threshold. This makes the model based on *M-d* more parsimonious because it fits only a single parameter, as opposed to two parameters for the model based on *d*. We find that the model based on *M-d* explains lock-in proneness better than the model based on *d*, according to a variety of metrics (log-likelihood: −10.55 versus –16.55; Akaike information criterion: 23.09 versus 37.1; Bayesian information criterion: 25.1 versus 41.11; McFadden’s $R^{2}$: 0.72 versus 0.56; *N* = 55; see table S4 for full estimation results). The results are similar in the case of kernel regression, which is nonparametric (log-likelihood: −6.74 versus –16.75; $R^{2}$: 0.86 versus 0.62; *N* = 55; see table S5). Thus, in both cases, *M-d* substantially outperforms *d* in explaining lock-in proneness.

The limited available data do not allow for a proper comparison of the out-of-sample predictive power of the two models for the following reason: Because lock-in is a probabilistic event, it is likely that in a small number of trials, no lock-in will be observed even when the process is actually lock-in-prone. Consequently, a model that confidently predicts that a process is lock-in-prone would often be severely penalized, even if this prediction is correct, while a model that makes the opposite, incorrect prediction will be favored. By reducing the number of trials that we use to assess the occurrence of lock-in, this phenomenon becomes much more common and thus makes any quantitative comparison unreliable.

Last, we emphasize that the relation M>d is not a post hoc condition obtained based on data fit, but one that we derived from first principles, assuming only that the choice probability is a function of an option’s popularity.

## DISCUSSION

Majority influence has been one of the main research topics in the study of social influence (*5*, *33*, *47*, *48*). The current conception of majority influence, as it has been also consolidated in formal models in the social and behavioral sciences, assumes that social influence gradually increases as the size of the majority increases (*7*, *37*–*39*). In this work, we defined and empirically demonstrated the marginal majority effect, a discontinuous increase in the choice probability of an option when it surpasses a competing option in popularity, and showed that it is a powerful mechanism of influence operating above and beyond the previously purported gradually increasing majority influence. In addition, we showed theoretically that when a large number of people choose from a given pair of options, the marginal majority effect provides a mechanism for lock-in. More precisely, we showed that lock-in may occur when the marginal majority effect exceeds the difference in inherent appeal of the two options (Theorem 2), and we obtained a lower bound for the probability that it occurs (Theorem 3). Last, we estimated the marginal majority effects and the differences in inherent appeal between options directly from data for more than 50 binary choice questions in three recent sequential choice experiments. We showed that our model explains the presence of lock-in in almost all cases, resolving a recent conundrum in the empirical literature.

### A qualitatively different type of lock-in

The marginal majority effect leads to a qualitatively different type of lock-in than previously identified. Past theoretical studies of lock-in have generally focused on systems that exhibit strong network externalities (*49*), such as in the context of competing technologies (*9*, *50*), coordination problems (*51*), and the formation of social norms (*52*). In the presence of network externalities, the value of choosing the more popular option is ever increasing with its popularity or, equivalently, the less popular option becomes increasingly less attractive as its popularity drops. The outcome is a spiraling feedback loop, reflected in the commonly used terms rich-get-richer dynamics or cumulative advantage, which are often associated with winner-take-all outcomes, i.e., with virtually everyone adopting the technology or social norm that wins the competition. In contrast, majorities of small magnitude (e.g., between 50 and 60%) are often thought to be unstable or transient, and they are theoretically impossible in many well-established models of sequential choice and cumulative advantage that predict winner-take-all outcomes (*6*, *10*, *11*). Here, we have shown theoretically and observed empirically that in the presence of marginal majority effects, small winning margins can be stable and become increasingly difficult to overturn over time, without necessarily leading to winner-take-all outcomes. We also pointed out that small, stable majorities of an inherently less attractive option are impossible for more classical models for which social influence is a gradually increasing function of popularity.

Another difference of the lock-in seen here from processes with network externalities is that in the latter case, as long as the competing options are of different quality, the critical mass is shifted in favor of the superior option, i.e., the critical mass of adopters of the superior option required to enter a regime where this option will dominate is smaller than 50% (*17*, *53*, *54*). Here, we find that if the process is lock-in-prone, then the critical mass, which in this framework can be precisely identified as the unstable equilibrium of the system (Fig. 1), is almost always located very close to 50% (Fig. 8) despite the two alternatives being of different inherent appeal. We note that while our theoretical analysis can be adapted to model abrupt changes in the degree of social influence at popularity values other than 50%, this would only make sense if there is good reason to focus at some other specific point. In the absence of a (psychological or structural) mechanism that renders a particular popularity value salient, the point of maximum sensitivity will necessarily vary with the specifics of the decision and contextual factors [see, e.g., (*54*)].

### The use of influence curves in studying social influence

Our theoretical and empirical demonstration of the marginal majority effect and its relation to lock-in relied on the concept of influence curves, a natural framework to study binary choice under social influence. The first use of influence curves that we are aware of dates back to Herbert Simon (*1*), who used them as a tool to understand the limits in the predictability of election polls. In the dynamic (sequential) stochastic choice setting, influence curves have been used to study the inheritance of cultural traits (*12*, *13*), technology adoption (*6*), opinion dynamics (*55*), and the evolution of online ratings (*56*). Similar frameworks, but with deterministic dynamics, are presented in (*30*–*32*). The deterministic case can be seen as a special case of the stochastic framework, where the influence curve of any given individual is a binary-valued function (see also section S1).

Within this framework, our work stands out in relating behavioral properties to the possibility of lock-in. Some early results in this direction appear in Granovetter’s seminal paper on threshold models (*30*), where he shows that a narrow distribution of individual adoption thresholds can prevent a behavior from spreading among people, thus locking the population in on nonadoption. More recently, Yang and colleagues (*28*) proposed a model in which they vary the proportion of social and asocial learners as well as the conformity bias of the social learners and explore the conditions under which such a system would be bistable. Other authors (*6*, *20*, *32*) make the distinction between systems with one or two stable equilibria but do not explore the behavioral properties that may put a system into one regime or the other. Similarly, models in the Bass diffusion tradition (*2*) primarily explain the rate at which an innovation diffuses through a market, not whether it can replace an incumbent product. Extensions that include competitive effects between innovations [(*56*); see also section 3.3 in (*57*)] still focus on diffusion dynamics rather than on the potential for bistability and lock-in studied here. Last, classic decision-theoretic models, such as the information cascade and herding model (*10*, *11*), are not suitable for studying the question of when lock-in may occur because in these models, lock-in is always possible by construction.

Influence curves have been previously reconstructed by other scholars using data from classic studies, such as the experiments by Asch (*5*), or new experiments designed exactly for that purpose (*19*, *58*–*62*). Here, we go a step further and use the reconstructed influence curves to test theoretical claims regarding the long-term behavior of a system, both those following from our theory and some that follow from previous theory, including the finding that the stable equilibria of an influence curve are the possible long-term values for popularity (*6*, *40*).

### Experimental settings and behavioral insights

As mentioned earlier, the role of marginal majorities in driving social influence has been largely ignored. In one of the experiments we analyzed (MDRT2019), participants were given information only about which of two popularity percentages was larger, not by how much; thus, marginal majority effects were structurally facilitated by limiting the provided information and the possible cognitive responses when making a choice. As Macy and colleagues point out [page 6 in (*18*)], providing only this binary information, rather than quantitative popularity information, can make it harder for a majority to be overturned because it prevents the progressive weakening of the popularity signal when participants choose the less popular option (until a large number of participants have made the same choice and the majority option changes). Our analysis here provides theoretical support for this observation. These structurally induced marginal majority effects may also be common in everyday experience, e.g., in media narratives reporting which candidate is currently leading in polls without providing detailed breakdowns of results.

In contrast to MDRT2019, in the V2019 and FV2021 datasets, the information about what the majority of previous participants had chosen was not emphasized (*20*, *24*). Even in these experiments, our analysis demonstrated the existence of sizable marginal majority effects for most of the questions, suggesting that marginal majorities might be psychologically salient even when they are not structurally emphasized [see also (*34*–*36*)]. One possible mechanism for this is if people use majorities as a simple social learning strategy when they feel uncertain about the correct answer or have not formed an opinion yet. There is evidence that people (and other animals) use simple adaptive strategies to guide their own decisions (*63*, *64*) and some evidence that they rely on the follow-the-majority strategy in particular (*65*). When even a fraction of individuals use such a heuristic strategy, a marginal majority effect is produced at the aggregate level (see section S1). In contrast, a marginal majority effect is incompatible with decision models that purport response thresholds that are sensitive to the number of individuals demonstrating a specific behavior, such as the quorum sensing models studied in animal behavior (*66*, *67*): Because a popularity of 50% corresponds to a different number of individuals depending on the total number of eligible individuals, there is no fixed threshold in terms of numbers that corresponds to it.

Deriving the influence curves of single individuals is empirically challenging, as it requires experiments designed to evoke multiple responses by the same individual to (near-)identical questions for various levels of popularity. However, it is possible to make inferences about individual behavior from aggregate patterns. Specifically, a marginal majority effect at the aggregate level guarantees the existence of a (near-)discontinuity of the response at the individual level for at least a subset of the population. The reason is that the aggregate influence curve is an average of the influence curves of the individuals in the population, and it is a mathematical fact that averaging preserves continuity. Hence, if all individuals had continuous influence curves, then the aggregate would also be continuous. More generally, averaging multiple influence curves may have only a smoothing effect, not produce a steeper curve.

In the FV2021 dataset, we found the marginal majority effect to be especially pronounced in the geometry questions, which were factual questions that required specific expertise, and, to a lesser extent, in the art trivia questions. The marginal majority effect was relatively smaller in the visual tasks questions, where prior knowledge was not needed. This is consistent with an adaptive information processing view of majority following (*33*, *68*) and with previous empirical work that finds that people are more likely to copy others or to conform when they have low confidence in their own judgment (*27*, *59*, *69*–*71*). Last, the marginal majority effects appear to be less pronounced in questions related to matters of taste, such as most of the questions in V2019, where the informational value of copying the majority is limited. In most of the questions in this study, people were asked which of two artifacts (e.g., paintings, screensavers, bowls, songs) they preferred. This suggests that for matters of taste the condition we identified will be satisfied only when the quality differences between the options are relatively small and that additional structural mechanisms might be needed to produce lock-in.

### Toward a general theory of popularity advantage

In the present study, we have focused on the case of two competing options. A generalization to multiple options should take into account not only relative majority (plurality) effects but also more general ranking effects, i.e., discontinuous changes in choice probabilities as an option exceeds in popularity another option in a ranking. The marginal majority effect can be seen as a special case of a ranking effect, specifically when there are only two options available.

A common framework for formalizing multinomial choice with reinforcement is preferential attachment models and their fitness-based extensions (*72*, *73*): The probability of gaining a new adopter is proportional to current popularity (degree) and intrinsic attractiveness (fitness). While this formalism captures popularity-based reinforcement, it does not take into account relative standing but only absolute degrees, so these models ignore ranking-based reinforcement.

At the opposite end of the spectrum, a mathematical framework for popularity dynamics of multiple options with purely ranking-based reinforcement has been developed in (*74*), showing that multiple equilibria may exist, and that the system is likely to converge to a suboptimal one (*75*). This framework applies to models and experimental settings in which choice probabilities depend only on the popularity ranking (*18*, *76*–*80*), an assumption that is fitting in settings where the exact popularity is not known (or ignored) by the individuals. This is indeed the case in many online interfaces such as search engines, recommender systems, and online marketplaces, where items are often displayed in a list ordered by their popularity. Ranking effects are especially pronounced in these systems because the display order of items heavily affects users’ search behavior and choice probabilities (*81*–*84*). The well-known Music Lab experiment (*3*) was also an early demonstration that ranking items by popularity can affect user behavior and the long-term popularity dynamics—in one of the experimental conditions, the songs were ranked by popularity for the users and this had an impact on the choice probabilities and overall outcomes.

An important challenge in dealing with more than two options when there are both ranking effects and continuously increasing popularity influence, as was the case in the Music Lab experiment, pertains to the state of current mathematical theory. Several authors have proposed computational models, sometimes accompanied with case-specific theoretical results (*85*–*89*), but currently no general mathematical theory exists. The mathematical approach we have used here treats the sequential choice process as a generalized urn problem (*40*, *41*). In the case of two competing options, the existence of one-dimensional influence curves (also known as “urn functions”) that characterize the system allows not only for a lot of intuition but also for the derivation of strong mathematical results (*40*, *90*). An approach that has been successfully applied to the study of generalized urn processes in any dimension is that of stochastic approximation theory (*91*–*94*). This framework can also incorporate discontinuous (e.g., ranking-driven) dynamics (*95*–*97*), so it could be leveraged to develop a general theory of the dynamics of popularity under social influence. Such a multidimensional “influence function theory” would allow researchers to study popularity dynamics under social influence in a more unified way, and to design multialternative multiple-world experiments that are suitable for studying the occurrence of lock-in, we thus consider it a promising avenue for future research.

## MATERIALS AND METHODS

### Empirical influence curves

V2019 and FV2021: For each question, we identify the inherently less appealing option (denoted as option *A*) as the option that was least popular in the control condition (no social influence). To estimate the influence curve, we first associate with each participant the “current popularity” of option *A*, that is the proportion of previous participants in the participant’s world (trial) that gave this answer. We then pool together participants from all worlds and separate them according to current popularity of *A* in bins of size 0.1. For each bin, we find the percentage of participants who chose *A*. The result is the value of the influence curve in that bin. Participants that fall on the bin end points are counted with weight one-half in each bin. In Figs. 6 and 8, participants that fall between the two central bins (*x* = 0.5) are excluded to highlight the marginal majority effect (definition of marginal majority effect does not include *x* = 0.5). See figs. S12 and S13 for the corresponding graphs with all participants. For V2019, apart from the trials reported in the main text, we use additional data from a trial reported in the appendix of (*20*), in which artificial counts were added between subsequent participants. Because these data were subjected to exogenous manipulations, it is used only in estimating the influence curves, not in identifying lock-in (see below).

MDRT2019: For each question, we identify as party *A* the party whose supporters were least likely to support the statement in the control condition. For each participant, we calculate the “current party *A* support” ($x_{A}$) and “current party *B* support” ($x_{B}$) separately, as the proportion of previous participants of the corresponding party that supported the statement in the participant’s world, and we define $x=x_{A}-x_{B}$. The responses of participants for which *x* = 0 are ignored when estimating the influence curves. The rest of the participants are grouped based on whether x>0 or x<0. The value of the influence curve for each group is estimated as $y=y_{A}-y_{B}$, where $y_{A}$ ($y_{B}$) is the percentage support of the statement among party *A* (*B*) supporters in that group.

### End-of-trial proportions and lock-in

V2019 and FV2021: For each question and each trial, the end-of-trial popularity of *A* ($\bar{y}$) is the proportion of participants in the trial that answered *A*. We say that there is a lock-in if $\bar{y}>0.5$ in at least one of the trials. MDRT2019: For each statement and each trial, we calculate the end-of-trial difference in support $\bar{y}={\bar{y}}_{A}-{\bar{y}}_{B}$, where ${\bar{y}}_{A}$ (${\bar{y}}_{B}$) is the proportion of party *A* (party *B*) supporters in the trial that supported the statement. We say that a lock-in occurs if $\bar{y}>0$ in at least one of the trials.

### Estimating marginal majority effects and difference in inherent appeal

V2019 and FV2021: We estimate the marginal majority effect as the increase in the value of the influence curve between the two central bins, i.e., from bin (0.4, 0.5) to (0.5, 0.6). The inherent appeal difference *d* is estimated as $p_{\text{ind}}^{B}-p_{\text{ind}}^{A}$, where $p_{\text{ind}}^{A}$ ($p_{\text{ind}}^{B}$) is the choice probability for *A* (*B*) in the control condition (no social influence).

MDRT2019: We define the marginal majority effect as $M=\frac{M_{A}-M_{B}}{2}$, where $M_{A}$ ($M_{B}$) is the marginal majority effect for supporters of party *A* (*B*), i.e., the difference in the probability of supporting a statement when this statement has larger percentage support by members of party *A* versus when it has larger support by members of party *B*. This definition makes sure that the maximum value for *M* is 1, and note that $M_{B}$ will normally be negative because the agreement probability for supporters of party *B* should be smaller when the statement has received more support by members of the other party (party *A*) as opposed to their own. Thus, M∈[0,1], which makes it comparable with the rest of the datasets.

The ideological content *d* of a statement is estimated as $d=p_{\text{ind}}^{B}-p_{\text{ind}}^{A}$, where $p_{\text{ind}}^{A}$ ($p_{\text{ind}}^{B}$) is the support probability for the statement among party *A* (*B*) supporters in the control condition. This can be justified as follows: Let $f_{A},f_{B}$ denote the influence curves for supporters of party *A* and *B*, respectively, and write

$$f_{A}\left(x\right)=g_{A}\left(x\right)+\frac{M_{A}}{2}\cdot u\left(x\right)$$

$$f_{B}\left(x\right)=g_{B}\left(x\right)+\frac{M_{B}}{2}\cdot u\left(x\right)$$

where $g_{A},g_{B}$ are continuous at *x* = 0, $M_{A},M_{B}$ are as above, and u(x)=±1 depending on whether x≷0 (see Eq. 1). The total influence curve $f\left(x\right)=f_{A}\left(x\right)-f_{B}\left(x\right)$ can thus be written as

f(x)=g(x)+M⋅u(x) (4)

where $g\left(x\right)=g_{A}\left(x\right)-g_{B}\left(x\right)$ and $M=\frac{M_{A}-M_{B}}{2}$ as above. Theorem 2 continues to hold if we take $d=\frac{d_{A}-d_{B}}{2}$, where $d_{A}=1-2\cdot g_{A}\left(0\right)$ is the difference between the probability of supporting and not supporting a statement for supporters of party *A* when the current percentage supports by the two parties are equal and similarly for $d_{B}$ (see the last sentence before Theorem 2). A natural estimate for $g_{A}\left(0\right)$ is $p_{\text{ind}}^{A}$ and similarly for $g_{B}\left(0\right)$. Since

$$d=\frac{d_{A}-d_{B}}{2}=\frac{\left[1-2\cdot g_{A}\left(0\right)\right]-\left[1-2\cdot g_{B}\left(0\right)\right]}{2}$$ (5)

$$=g_{B}\left(0\right)-g_{A}\left(0\right)$$ (6)

we may estimate *d* by $p_{\text{ind}}^{B}-p_{\text{ind}}^{A}$.

### Logistic and kernel regression and goodness-of-fit for lock-in proneness

For the evaluation of *d* and *M-d* as regressors of lock-in proneness, we applied both logistic and Nadaraya-Watson kernel regression. For the logistic regression we used the built-in function of Python’s statsmodels package (v0.14.5), with *d* or *M-d* as the only regressor (plus intercept in the case of *d*). The Nadaraya-Watson kernel regression [section 4.1.1 in (*98*)] is a nonparametric method that, when applied to binary data, gives an estimate of ℙ (Y=1∣X=x), where *X* is a continuous regressor, based on observations that fall around *x* and weighted according to their distance from *x*, with more distant points receiving smaller weights. We used the Python package statsmodels implementation of this estimator, which uses a Gaussian kernel (weighting function) with bandwidth (SD) optimized based on a least squares leave-one-out cross-validation criterion. The $R^{2}$ metric denotes the square of the correlation coefficient between the vector of fitted probabilities and the vector of true (binary) outcomes [see section 4.3 in (*98*)].

### Confidence intervals for number of lock-in occurrences

To obtain confidence intervals and *P* values for the number of lock-in occurrences for individual experimental items, we used the stats.binom module from Python package scipy (v1.16.2), with probabilities obtained from Eq. 2. For the aggregate number of lock-in occurrences in all items that satisfied M>d, we simulated 10^5^ Poisson binomial random variables using the built-in function stats.poisson_binom.rvs of the same Python package, with parameter vector equal to the list of lock-in probabilities obtained from Eq. 2 for those items, each with multiplicity equal to the number of trials of the corresponding item. The lower bound of the 95% one-sided confidence interval was calculated as the 5000th smallest value obtained.

### Proofs of theorems

Here, we prove Theorems 1 to 3. In what follows, *I* and *J* denote intervals of real numbers (we may take I=J=[0,1]). We use the definition of crossing/downcrossing the diagonal from (*40*). The assumptions on *f* stated before Theorem 1 should be understood with respect to this definition.

Definition 2. Let f:I→J be any map. We say that *f* crosses the diagonal at $x_{0}\in I$ if for every ϵ>0, there exist points $x_{1},x_{2}\in \left(x_{0}-\epsilon ,x_{0}+\epsilon \right)$, such that $f\left(x_{1}\right)>x_{1}$ and $f\left(x_{2}\right)<x_{2}$.

We say that *f* downcrosses the diagonal at $x_{0}\in I$ if there exists some ϵ>0, such that f(x)>x for all $x\in \left(x_{0}-\epsilon ,x_{0}\right)$ and f(x)<x for all $x\in \left(x_{0},x_{0}+\epsilon \right)$.

Before we proceed to the proofs of our theorems, we need a couple of lemmas.

Lemma 1. Let f:I→J be an increasing function and let a,b∈I, a<b, be such that f(a)>a and f(b)<b. Then, there exists some c∈(a,b) such that f(c)=c.

If *f* was assumed to be continuous, then the above statement would follow immediately from the intermediate value theorem. Here, we are not assuming continuity for *f*.

**Proof:** Let c=inf{x>a:f(x)<x}. The assumption f(b)<b implies that c≤b, while by definition, we have c≥a. We claim that f(c)=c, which will also imply that c≠a,b; hence, c∈(a,b).

Suppose on the contrary that f(c)≠c. If f(c)<c, then $x=\frac{c+f\left(c\right)}{2}$ has the property that x<c, and since *f* is increasing, $f\left(x\right)\leq f\left(c\right)<\frac{c+f\left(c\right)}{2}=x$, which contradicts the definition of *c*. If f(c)>c, then for any $x\in \left[c,f\left(c\right)\right)$ we have f(x)≥f(c)>x; hence, no element of $\left[c,f\left(c\right)\right)$ is in the set {x>a:f(x)<x}. This again contradicts the definition of *c* as the infimum of the latter set. We conclude that f(c)=c, which completes the proof. □

Lemma 2. Let f:I→J be an increasing function, and suppose that it meets the diagonal at most finitely many times. Let a,b∈I be such that a<b, f(a)>a, and f(b)<b. Then, *f* downcrosses the diagonal at some x∈(a,b).

**Proof:** By Lemma 1, f(x)=x has at least one solution, and by assumption, it has finitely many solutions. Let $x_{1}<\ldots <x_{k}$ be these solutions. The quantity *f*(*x*) − *x* cannot change sign in any interval of the form $\left(x_{i},x_{i+1}\right)$ because then Lemma 1 would imply that there is an extra solution of f(x)=x in that interval. For similar reasons, *f*(*x*) − *x* is positive on $\left(a,x_{1}\right)$ and negative on $\left(x_{k},b\right)$. Therefore, *f*(*x*) − *x* has constant sign on each of the intervals $\left(a,x_{1}\right),\left(x_{1},x_{2}\right),\ldots ,\left(x_{k-1},x_{k}\right),\left(x_{k},b\right)$. Because this sign is positive on $\left(a,x_{1}\right)$ and negative on $\left(x_{k},b\right)$, we conclude that there exists some *i*, such that *f*(*x*) − *x* is positive on $\left(x_{i-1},x_{i}\right)$ and negative on $\left(x_{i},x_{i+1}\right)$ (where we define $x_{0}=a$ and $x_{k+1}=b$), making $x_{i}$ a downcrossing. □

Apart from the above lemmas, the Proof of theorem 1 relies on two results from (*40*): (i) The process may only converge to a point where *f* crosses the diagonal [Proposition 3.1 in (*40*)], and (ii) for any point where *f* downcrosses the diagonal, there is positive probability that the process converges to it [Theorem 4.2 in (*40*)].

Proof of theorem 1: Suppose first that *f* enters the lock-in region, that is, there is some a>0.5 such that f(a)>a. By our assumptions on *f*, we have f(1)<1 (see paragraph before statement of Theorem 1). Hence, by Lemma 2, *f* downcrosses the diagonal at some $x^{*}\in \left(a,1\right)\subset \left(0.5,1\right)$. By Theorem 4.2 in (*40*), $\mathbb{P}\left(x\to x^{*}\right)>0$.

For the converse, let S={x∈[0,1]:f(x)<x} and denote by $S^{o}$ its interior. By Proposition 3.1b in (*40*), we have $\mathbb{P}\left(x_{\infty }\in S^{o}\right)=0$. If the process is lock-in-prone, i.e., $\mathbb{P}\left(x_{\infty }>0.5\right)>0$, then $\left(0.5,1\right]⊄S^{o}$; hence, there exists some $x^{*}>0.5$, such that $f\left(x^{*}\right)\geq x^{*}$. If $f\left(x^{*}\right)>x^{*}$, then this concludes the proof. If $f\left(x^{*}\right)=x^{*}$, then by our assumption that *f* crosses the diagonal whenever it meets it, *f* must enter the lock-in region either directly below or directly above $x^{*}$ (more precisely, arbitrarily close to $x^{*}$), again concluding the proof. □

Proof of theorem 2: The right-sided limit of *f* at *x* = 0.5 is

$$c=\underset{x\to 0.5^{+}}{\text{lim}}\;f\left(x\right)=\underset{x\to 0.5^{+}}{\text{lim}}\;\left[g\left(x\right)+\frac{M}{2}\cdot u\left(x\right)\right]=g\left(0.5\right)+\frac{M}{2}$$ (7)

because *g* is continuous at *x* = 0.5 and u(x)=1 for any x>0.5. The assumption M>d=1−2⋅g(0.5) can be written as M/2+g(0.5)>1/2; hence, we get c>1/2. Since *f* is increasing, for any x∈(0.5,c), we have $f\left(x\right)\geq \underset{x\to 0.5^{+}}{\text{lim}}\;f\left(x\right)=c>x$; hence, *f* enters the lock-in region. The result now follows from Theorem 1. □

The proof in the case that *f* is given through Eq. 4, with the definitions of *g*(*x*), *M*, *u*(*x*), and *d* that follow it, is completely analogous. Note that an analog of Theorem 1 is also required, which in the case where *f* is constant on x>0 and on x<0 follows from Corollary 2.11 in (*74*).

For the Proof of theorem 3, we will also need a lemma.

Lemma 3. Let $X_{n}$ be a Markov chain on ℤ, taking unit steps, i.e., $X_{n+1}-X_{n}\in \left\{-1,1\right\}$ for any *n*. Let $q_{k}$ denote the probability of taking a step to the right when the process is at state *k*, i.e., $q_{k}=\mathbb{P}\left(X_{n+1}-X_{n}=1|X_{n}=k\right)$, and suppose that $q_{k}=q_{+}>0.5$ for any k>0. Then,

$$\mathbb{P}(X_{n}\geq 0\;\text{for}\;\text{all}\;n\in \mathbb{N}|X_{0}=0)=\frac{q_{0}\cdot \left(2q_{+}-1\right)}{q_{+}-q_{0}\left(1-q_{+}\right)}$$ (8)

**Proof:** First, consider the probability $p_{1}$ that, given that the process is at state *k* = 1, it never visits 0, i.e., $p_{1}=\mathbb{P}\left(X_{n}>0\;\text{for}\;\text{all}\;n>n_{0}|X_{n_{0}}=1\right)$, which by time homogeneity satisfies $p_{1}=\mathbb{P}\left(X_{n}>0\;\text{for}\;\text{all}n>0|X_{0}=1\right)$. Because on states k>0, the probabilities of moving to the right or left are constant, in calculating $p_{1}$, we may treat $X_{n}$ as a homogeneous (biased) random walk, with probability of moving to the right equal to $q_{+}$. For such a random walk, with $q_{+}>0.5$ and $X_{0}=1$, the probability of never visiting 0 is given by $p_{1}=1-\frac{1-q_{+}}{q_{+}}=\frac{2q_{+}-1}{q_{+}}$ [see example 1.54 in (*99*)].

We denote the left-hand side of Eq. 8 by *x*. For the chain never to become negative, the first step must be to the right; therefore

$$x=q_{0}\cdot \mathbb{P}\left(X_{n}\geq 0\;\text{for}\;\text{all}\;n>0|X_{1}=1\right)$$ (9)

Let τ denote the first time *X*_n_ visits 0, after *n* = 0 (and set τ=∞ if this never happens). The last factor in the above equation can be decomposed as follows

$$\begin{array}{c} \mathbb{P}\left(X_{n}\geq 0\;\text{for}\;\text{all}\;n>0|X_{1}=1\right)=\\ \mathbb{P}\left(X_{n}>0\;\text{for}\;\text{all}\;n>0|X_{1}=1\right)+\\ \sum_{k=2}^{\infty }\mathbb{P}\left(\tau =k|X_{1}=1\right)\cdot \mathbb{P}\left(X_{n}\geq 0\;\text{for}\;\text{all}\;n\geq \tau |\tau =k\right)=\\ p_{1}+\sum_{k=2}^{\infty }\mathbb{P}\left(\tau =k|X_{1}=1\right)\cdot \mathbb{P}\left(X_{n}\geq 0\;\text{for}\;\text{all}\;n\geq \tau |\tau =k\right) \end{array}$$ (10)

Because by definition $X_{\tau }=0$ whenever τ is finite, by the Markov property, we have

$$\begin{array}{c} \mathbb{P}\left(X_{n}\geq 0\;\text{for}\;\text{all}\;n\geq \tau |\tau =k\right)=\mathbb{P}\left(X_{n}\geq 0\;\text{for}\;\text{all}\;n\geq \tau |X_{\tau }=0\right)\\ =\mathbb{P}\left(X_{n}\geq 0\;\text{for}\;\text{all}\;n\geq 0|X_{0}=0\right)\\ =x \end{array}$$ (11)

where the second equality follows from time homogeneity. Substituting this into Eq. 10, we get

$$\mathbb{P}\left(X_{n}\geq 0\;\text{for}\;\text{all}\;n>0|X_{1}=1\right)=p_{1}+x\cdot \sum_{\tau =2}^{\infty }\mathbb{P}\left(\tau =k|X_{1}=1\right)$$ (12)

Now observe that $\sum_{\tau =2}^{\infty }\mathbb{P}\left(\tau =k|X_{1}=1\right)$ is the total probability of visiting 0 at some point, given that $X_{1}=1$, which is the complement of what we have denoted by $p_{1}$. Therefore, the above equation becomes

$$\mathbb{P}\left(X_{n}\geq 0\;\text{for}\;\text{all}\;n>0|X_{1}=1\right)=p_{1}+x\cdot \left(1-p_{1}\right)$$ (13)

Now substituting this into Eq. 9, we get

$$x=q_{0}\cdot \left[p_{1}+x\cdot \left(1-p_{1}\right)\right]\Leftrightarrow x=\frac{q_{0}\cdot p_{1}}{1-q_{0}\left(1-p_{1}\right)}$$ (14)

Last, recall that $p_{1}=\frac{2q_{+}-1}{q_{+}}$; hence

$$x=\frac{q_{0}\cdot \frac{2q_{+}-1}{q_{+}}}{1-q_{0}\cdot \left(1-\frac{2q_{+}-1}{q_{+}}\right)}=\frac{q_{0}\cdot \left(2q_{+}-1\right)}{q_{+}-q_{0}\cdot \left(1-q_{+}\right)}$$ (15)

which completes the proof. □

Proof of theorem 3: Let $Y_{n}$ denote the number of times that option *A* has been chosen among the first *n* agents. The popularity of *A* is $Y_{n}/n$, and $x_{\infty }=\underset{n\to \infty }{\text{lim}}\;Y_{n}/n$. Recall that the influence curve for this binary choice process can be written as $f\left(x\right)=g\left(x\right)+\frac{M}{2}\cdot u\left(x\right)$, where *g* is continuous, and that we have the relation $d=1-2g\left(0.5\right)\Leftrightarrow g\left(0.5\right)=\frac{1-d}{2}$. Let $c=\underset{x\to 0.5^{+}}{\text{lim}}\;f\left(x\right)$ as in the Proof of theorem 1. Because *f* is nondecreasing, we have f(x)≥c for any x>0.5. Moreover, by Eq. 7, we have $c=g\left(0.5\right)+\frac{M}{2}=\frac{1-d+M}{2}>0.5$.

Now consider an alternative binary choice process with influence curve given by

$$\hat{f}\left(x\right)=\left\{\begin{array}{cc} \frac{1-d+M}{2}, & \text{if}\;x>0.5,\\ \frac{1-d}{2}, & \text{if}\;x=0.5,\\ 0, & \text{if}\;x<0.5, \end{array}\right.$$ (16)

and denote by ${\hat{Y}}_{n}$ the number of times option *A* has been chosen up to agent *n*. By construction, we have that $\hat{f}\left(x\right)\leq f\left(x\right)$ for all *x*; therefore

$$\mathbb{P}\left(x_{\infty }>0.5\right)=\mathbb{P}\left(\underset{n\to \infty }{\text{lim}}\;Y_{n}/n>0.5\right)\geq \mathbb{P}\left(\underset{n\to \infty }{\text{lim}}\;{\hat{Y}}_{n}/n>0.5\right)$$ (17)

It is thus enough to show that the required bound holds for $\mathbb{P}\left(\underset{n\to \infty }{\text{lim}}\;{\hat{Y}}_{n}/n>0.5\right)$.

Since we know that ${\hat{Y}}_{n}/n$ converges to one of the stable equilibria of $\hat{f}$, which lie at $x=\frac{1-d+M}{2}>0.5$ and *x* = 0, the quantity $\mathbb{P}\left(\underset{n\to \infty }{\text{lim}}\;{\hat{Y}}_{n}/n>0.5\right)$ is equal to the probability that for all sufficiently large *n*, we have ${\hat{Y}}_{n}/n\geq 0.5$. A stricter condition is to require that ${\hat{Y}}_{n}/n\geq 0.5$ for all n∈ℕ; therefore

$$\mathbb{P}\left(\underset{n\to \infty }{\text{lim}}\;{\hat{Y}}_{n}/n>0.5\right)\geq \mathbb{P}\left({\hat{Y}}_{n}/n\geq 0.5\;\text{for}\;\text{all}\;n\in \mathbb{N}\right)$$ (18)

(Note that $\underset{n\to \infty }{\text{lim}}\;{\hat{Y}}_{n}/n>0.5$ is equivalent to $\underset{n\to \infty }{\text{lim}}\;{\hat{Y}}_{n}/n\geq 0.5$ because we know that there is no stable equilibrium at *x* = 0.5). We will obtain an expression for the right-hand side.

Let $X_{n}=2{\hat{Y}}_{n}-n$ and observe that $X_{n}$ is a process taking values on the integers and that, at each step, $X_{n}$ either increases or decreases by *1*, depending on whether ${\hat{Y}}_{n}$ increases by 1 or remains constant. That is, $X_{n+1}-X_{n}\in \left\{-1,1\right\}$ and $X_{n+1}-X_{n}=1$ if and only if ${\hat{Y}}_{n+1}-{\hat{Y}}_{n}=1$. The probability of the latter event is by definition equal to $\hat{f}\left({\hat{Y}}_{n}/n\right)=\hat{f}\left(\frac{X_{n}}{2n}+\frac{1}{2}\right)$. Therefore

$$\begin{array}{c} \mathbb{P}\left(X_{n+1}-X_{n}=1|X_{n}=k\right)=\hat{f}\left(\frac{k}{2n}+\frac{1}{2}\right)=\left\{\begin{array}{cc} \frac{1-d+M}{2}, & \text{if}\;\frac{k}{2n}+\frac{1}{2}>\frac{1}{2}\\ \frac{1-d}{2}, & \text{if}\;\frac{k}{2n}+\frac{1}{2}=\frac{1}{2}\\ 0, & \text{if}\;\frac{k}{2n}+\frac{1}{2}<\frac{1}{2} \end{array}\right.\\ =\left\{\begin{array}{cc} \frac{1-d+M}{2}, & \text{if}\;k>0\\ \frac{1-d}{2}, & \text{if}\;k=0\\ 0, & \text{if}\;k<0 \end{array}\right. \end{array}$$ (19)

Therefore, $X_{n}$ satisfies the conditions of Lemma 3 with $q_{+}=\frac{1-d+M}{2}$ and $q_{0}=\frac{1-d}{2}$; thus

$$\begin{array}{cc} \mathbb{P}\left(X_{n}\geq 0\;\text{for}\;\text{all}\;n\in \mathbb{N}|X_{0}=0\right) & =\frac{q_{0}\cdot \left(2q_{+}-1\right)}{q_{+}-q_{0}\cdot \left(1-q_{+}\right)}\\ & =\frac{\frac{1-d}{2}\cdot \left(M-d\right)}{\frac{1-d+M}{2}-\frac{1-d}{2}\cdot \frac{1-M+d}{2}}\\ & =\frac{2\left(1-d\right)\left(M-d\right)}{2\left(1+M-d\right)-\left(1-d\right)\left(1-M+d\right)}\\ & =\frac{2\left(1-d\right)\left(M-d\right)}{2M+\left(1-d\right)\left(1+M-d\right)} \end{array}$$ (20)

Moreover, we have that $X_{0}=2{\hat{Y}}_{0}-0=0$ by assumption, while $X_{n}\geq 0$ is equivalent to ${\hat{Y}}_{n}/n\geq \frac{1}{2}$. Therefore, the above equation becomes

$$\mathbb{P}\left({\hat{Y}}_{n}/n\geq 1/2\;\text{for}\;\text{all}\;n\in \mathbb{N}\right)=\frac{2\left(1-d\right)\left(M-d\right)}{2M+\left(1+d\right)\left(1+M-d\right)}$$ (21)

In view of Eqs. 17 and 18, this concludes the proof. □

We note that Theorem 3 does not hold strictly for the MDRT2019 dataset, where the participants had information about which of two percentages was larger (democrat or republican support), rather than the value of a single percentage quantity. However, the assertion continues to hold under the assumption that the number of democrat and republican participants is equal throughout the experiment (see remark after the proof of theorem S1 in the Supplementary Materials), which would be the case only if democrat and republican participants alternated. Since the order of the participants in MDRT2019 was random, this assumption is only approximately satisfied.

## Supplementary Materials

### This PDF file includes:

Sections S1 to S5 Figs. S1 to S13 Tables S1 to S6
