# Supplementary material for: The marginal majority effect: When social influence produces lock-in
Source: Sci Adv. 2026 Feb 18;12(8):eadr4237. doi: 10.1126/sciadv.adr4237 (PMC12915603; doi:10.1126/sciadv.adr4237)
Supplement: Supplementary file 1 — Sections S1 to S5 Figs. S1 to S13 Tables S1 to S6 [file sciadv.adr4237_sm.pdf]

Science Advances
AAAS

Supplementary Materials for

The marginal majority effect: When social influence produces lock-in

Alexandros Gelastopoulos *et al.*

Corresponding author: Alexandros Gelastopoulos, alexandros.gelastopoulos@iast.fr

Sci. Adv. **12**, eadr4237 (2026)
DOI: 10.1126/sciadv.adr4237

This PDF file includes:

Sections S1 to S5
Figs. S1 to S13
Tables S1 to S6

1 From individual to aggregate influence curves

Influence curves as used in this paper are an aggregate-level theoretical construct, produced by averaging behavioral tendencies and data across individuals. Influence curves, however, can also be conceived at the individual level, expressing the extent to which different individuals are influenced by others in specific contexts and the behavioral strategies they might follow (see Fig. S1). For example, in the quiz experiments reported in FV2021, some people might be certain that they know the answer, which means that their influence curves will be unresponsive to social influence (Fig. S1, top left). Others may be completely ignorant of the answer and use a proportional response or a follow-the-majority strategy (Fig. S1 top right and bottom left, respectively). Yet other individuals might have some background knowledge without being certain about the answer, and thus might be influenced in an arbitrary non-linear way by what other people have responded (Fig. S1, bottom right).

If the influence curves of individuals are known, they can be aggregated through averaging in order to derive the population-level influence curve $f(x)$. The latter can be used to predict the possibility of lock-in, under the assumption of random sampling (see Background and definitions section of the main text). For example, Granovetter's famous threshold model [1] assumes that people's individual-level curves are step functions, jumping discontinuously from probability 0 to 1 (similar to Fig. S1, bottom left), but that the thresholds in the population vary. That is, some individuals could be triggered to act even if a small proportion of others did, whereas others may require a larger established base of other people acting before they do so themselves. When aggregated, these discontinuous step functions will often produce a smooth, continuously increasing function (e.g., if the thresholds are normally distributed). The aggregate curve will have discontinuities if there is a positive fraction of the population with the same threshold, which may occur, for example, if a subset of the population adopts the follow-the-majority heuristic.

In practice, empirical data often allows one to derive directly the aggregate influence curve, but not the individual-level ones. Deriving individual-level curves would require empirical observations at different levels of social influence for a single individual in the same exact social influence setting, which in most cases is hard to obtain.

2 Exact probability of lock-in for influence curves that are step functions

Theorem 3 in the main text gives a lower bound for the lock-in probability that applies to any (non-decreasing) influence curve. Our empirical results showed that lock-in occurs substantially more often than this minimum, thus satisfying the theoretical predictions, but we had no way to say whether lock-in occurred too often. Here we obtain a sharper result for the case that the influence curve is a step function of the form $f(x) = a + \frac{M}{2} \cdot u(x)$, where a and M are constants, $u(x) = 1$ or -1 depending on whether $x > 0.5$ or $x < 0.5$, and $u(0.5) = 0$. Specifically, we find the *exact* lock-in probability for influence curves of this form, which allows us to derive a two-sided confidence interval for the number of trials that exhibit lock-in out of a given total number of trials. We then apply this result to the MDRT2019 dataset (which satisfies the assumptions of the theorem *approximately*—see Remark S3 below) and find that the empirically observed number of lock-in occurrences agrees with the theoretical predictions.

Theorem S1. Let $f(x) = a + \frac{M}{2} \cdot u(x)$. If $M > d := 1 - 2a$, then the lock-in probability is

$$p_L := \mathbb{P}\left(x_\infty > 0.5\right) = \frac{(M-d)(1+M+d)(1-d)}{2M(1+M)}. \tag{S1}$$

As an example, if $M = 0.2$ and $d = 0.1$, we get $p_L = \frac{(0.2-0.1)(1+0.2+0.1)(1-0.1)}{2 \cdot 0.2(1+0.1)} = \frac{0.117}{0.44} \approx$ 26.6%, which is twice as large as the lower bound obtained by theorem 3 in the main text.

Table S1 shows the 95% confidence interval for the number of lock-in occurrences in 8 trials for each of the items in MDRT2019, based on the lock-in probabilities obtained from Eq. (S1), along with the empirical number of trials that exhibited lock-in. These confidence intervals were obtained as described in the Methods section of the main text (except that here we use Eq. (S1) and two-sided confidence intervals). Despite the fact that the experimental setup satisfies the conditions for Theorem S1 only approximately (see Remark S3), the empirical outcomes lie in the theoretical 95% confidence intervals for each item separately, as well as for the aggregate number of lock-in occurrences for all items together. These results provide moderate to strong support for the explanatory power of Theorem S1 for the frequency of lock-in.

We now return to the proof of Theorem S1. We will need the following lemma.

Lemma S2. Let X_n be a Markov chain on $\mathbb{Z}$, with $X_0 = 0$ and such that $X_{n+1} - X_n \in \{-1, 1\}$ for all $n \in \mathbb{N}$. Suppose that

$$\mathbb{P}\left(X_{n+1} - X_n = 1 \mid X_n = k\right) = \begin{cases} q_+, & \text{if } k > 0 \\ q_0, & \text{if } k = 0 \\ q_-, & \text{if } k < 0 \end{cases}, \tag{S2}$$

for some positive constants q_+, q_-, and q_0 such that $q_- < 0.5 < q_+$. Let A^+ denote the event that after some point in time, X_n stays strictly on the positive integers, i.e.,

$$A^+ = \{\exists n_0 \in \mathbb{N} : X_n > 0 \text{ for all } n > n_0\}. \tag{S3}$$

Then,

$$\mathbb{P}\left(A^+\right) = \frac{(2q_+ - 1)(1 - q_-)q_0}{(2q_+ - 1)(1 - q_-)q_0 + (1 - q_0)(1 - 2q_-)q_+} \tag{S4}$$

This is essentially Proposition 3.3 in [2], with slightly different notation. Here we only sketch the proof.

Proof sketch for Lemma S2. Let p_+ and p_- denote the probabilities that the first step of the random walk is to the right, respectively left, and that it never returns to 0. p_+ can be written as the product of the probability that the first step is to the right ($X_1 = 1$) and the conditional probability that X_n never returns to 0 given that $X_1 = 1$, that is $p_+ = q_0 \cdot p_1$, where $p_1 = \mathbb{P}\left(X_n > 0 \text{ for all } n > 0 \mid X_1 = 1\right) = \frac{2q_+ - 1}{q_+}$ (see proof of theorem 3 in Appendix B of the main text). Thus, $p_+ = \frac{q_0 \cdot (2q_+ - 1)}{q_+}$ and similarly we get $p_- = \frac{(1 - q_0) \cdot (1 - 2q_-)}{1 - q_-}$.

Since the total probability of never returning to 0 (i.e., $p_+ + p_-$) is positive, 0 is a transient state, implying that X_n eventually stops returning to 0 with probability 1.

Let τ denote the *last* time that X_n visits 0. By time-homogeneity, there is no loss of generality in assuming that $\tau = 0$. In other words, in calculating the probability of A^+, we may condition on the event that X_n never visits 0 after $n = 0$. Then the probability of A^+ equals the probability that the first step is to the right. That is,

$$\begin{aligned} \mathbb{P}\left(A^+\right) &= \mathbb{P}\left(X_n = 1 \mid X_n \neq 0 \text{ for all } n > 0\right) \\ &= \frac{\mathbb{P}\left(X_n = 1 \text{ and } X_n \neq 0 \text{ for all } n > 0\right)}{\mathbb{P}\left(X_n \neq 0 \text{ for all } n > 0\right)} \\ &= \frac{p_+}{p_+ + p_-}. \end{aligned} \tag{S5}$$

The result now follows from substituting the values of p_+ and p_- into the last equation. □

Proof of Theorem S1. Let Y_n denote the number of times that option A has been chosen among the first n agents. The popularity of A is Y_n/n. Since we know that Y_n/n converges to

one of the stable equilibria of the influence curve, which are at $x^+ = a + M/2 > 1/2$ and $x^- = a - M/2 < 1/2$, the probability of lock-in is equal to the probability that Y_n/n will eventually take values only above 1/2. In other words,

$$\mathbb{P}(x_\infty > 0.5) = \mathbb{P}(\exists n_0 \in \mathbb{N} : Y_n/n > 0.5 \text{ for all } n > n_0). \tag{S6}$$

Define $X_n = 2Y_n - n$. We have that $X_n > 0 \Leftrightarrow Y_n/n > 0.5$, hence we can rewrite Eq. (S6) as

$$\begin{aligned}\mathbb{P}(x_\infty > 0.5) &= \mathbb{P}(\exists n_0 \in \mathbb{N} : X_n > 0 \text{ for all } n > n_0) \\ &= \mathbb{P}\left(A^+\right),\end{aligned} \tag{S7}$$

where A^+ is given by Eq. (S3). Moreover, $X_{n+1} - X_n \in \{-1, 1\}$ and $X_{n+1} - X_n = 1$ if and only if $Y_{n+1} - Y_n = 1$, which occurs by definition with probability $f(Y_n/n) = f\left(\frac{X_n}{2n} + \frac{1}{2}\right)$ (see also the proof of theorem 3 in the main text). Therefore,

$$\begin{aligned}\mathbb{P}(X_{n+1} - X_n = 1 \mid X_n = k) = f\left(\frac{k}{2n} + \frac{1}{2}\right) &= \begin{cases} a + \frac{M}{2}, & \text{if } \frac{k}{2n} + \frac{1}{2} > \frac{1}{2} \\ a, & \text{if } \frac{k}{2n} + \frac{1}{2} = \frac{1}{2} \\ a - \frac{M}{2}, & \text{if } \frac{k}{2n} + \frac{1}{2} < \frac{1}{2} \end{cases} \\ &= \begin{cases} \frac{1-d+M}{2}, & \text{if } k > 0 \\ \frac{1-d}{2}, & \text{if } k = 0 \\ \frac{1-d-M}{2}, & \text{if } k < 0 \end{cases},\end{aligned} \tag{S8}$$

where we have used the fact that $d = 1 - 2a \Leftrightarrow a = \frac{1-d}{2}$. Therefore, Lemma S2 applies with $q_+ = \frac{1-d+M}{2} > 1/2$, $q_0 = \frac{1-d}{2}$ and $q_- = \frac{1-d-M}{2} < 1/2$ and gives

$$\begin{aligned}\mathbb{P}\left(A^+\right) &= \frac{(2q_+ - 1)(1 - q_-)q_0}{(2q_+ - 1)(1 - q_-)q_0 + (1 - q_0)(1 - 2q_-)q_+} \\ &= \frac{(M - d) \cdot \frac{1+d+M}{2} \cdot \frac{1-d}{2}}{(M - d) \cdot \frac{1+d+M}{2} \cdot \frac{1-d}{2} + \frac{1+d}{2} \cdot (M + d) \cdot \frac{1-d+M}{2}} \\ &= \frac{(M - d) \cdot (1 + d + M) \cdot (1 - d)}{(M - d) \cdot (1 + d + M) \cdot (1 - d) + (1 + d) \cdot (M + d) \cdot (1 - d + M)}.\end{aligned} \tag{S9}$$

The denominator can be simplified. We expand with respect to powers of M:

$$\begin{aligned}&(M - d) \cdot (1 + d + M) \cdot (1 - d) + (1 + d) \cdot (M + d) \cdot (1 - d + M) \\ &= M^2 \cdot [(1 - d) + (1 + d)] \\ &+ M \cdot [(1 + d)(1 - d) - d(1 - d) + (1 + d)(1 - d) + (1 + d)d] \\ &+ [-d(1 + d)(1 - d) + (1 + d)d(1 - d)] \\ &= 2M^2 + 2M \\ &= 2M(M + 1).\end{aligned} \tag{S10}$$

Substituting this into Eq. (S9) and recalling that $\mathbb{P}(x_\infty > 0.5) = \mathbb{P}\left(A^+\right)$, we obtain Eq. (S1). □

Remark S3. Recall that the experimental setting of MDRT2019 differs from the base model considered in this paper, because the information that participants received did not reflect the percentage support of a statement, but how two percentages (agreement with a statement among democrats and among republicans) compared to each other. While theorems 1 and 2 of the main text also applied to this case, with suitable definitions of the influence curve and of the variables M and d (see the main text), this is not exactly the case for Theorem S1 or for theorem 3 in the main text. The assertion of Theorem S1 would still hold if the number of democrat and republican participants remained equal throughout the experiment, which would

be the case only if democrat and republican participants alternated. Since the order of the participants in MDRT2019 was random, this assumption is only approximately satisfied.

To prove the claim that the theorem assertion holds if democrat and republican participants remain equal throughout the experiment, consider first a scenario in which (i) every participant is equally likely to be republican or democrat (which is approximately true in the MDRT2019 experiment; 48% of the participants were democrat supporters) and (ii) the information the participants have is whether so far more democrats or republicans agreed more with a statement in terms of *absolute* numbers, rather than percentages. If we let $X_n = Y_n^A - Y_n^B$ denote the difference in the number of party A and party B supporters who agreed with a statement up to participant n, then under the above assumptions X_n would increase by 1 if the next participant was a party A supporter and agreed with the statement or a party B supporter who disagreed with the statement, and it would decrease by 1 otherwise. Thus, $\mathbb{P}\left(X_{n+1} - X_n = 1 \mid X_n > 0\right) = 0.5 \cdot q_A^A + 0.5 \cdot (1 - q_A^B)$, where q_j^i denotes the probability of agreeing with the statement for a party i supporter, given that currently there is more support by party j's supporters. Substituting $q_A^A = \frac{1-d_A+M_A}{2}$ and $q_A^B = \frac{1-d_B+M_B}{2}$, we get

$$\begin{aligned}\mathbb{P}\left(X_{n+1} - X_n = 1 \mid X_n = k\right) &= 0.5 \cdot \frac{1 - d_A + M_A + 1 - d_B + M_B}{2} \\ &= \frac{1 - M + d}{2}\end{aligned} \tag{S11}$$

if $k > 0$ and similarly we obtain $\frac{1-d-M}{2}$ if $k < 0$ and $\frac{1-d}{2}$ if $k = 0$. These are the exact same values as Eq. (S8), and the rest of the proof remains the same.

In the MDRT2019 experiment, the information participants had was in terms of percentage support, i.e., whether $X_n' = \frac{Y_n^A}{k_n^A} - \frac{Y_n^B}{k_n^B}$ was positive or negative, where k_n^A and k_n^B denote the number of supporters of parties A and B among the first n participants. Because the order of the participants was random, $k_n^A \neq k_n^B$ in general. However, if we assume that $k_n^A \approx k_n^B$, then $X_n' > 0$ is equivalent to $X_n > 0$, so Theorem S1 should give an approximate probability of lock-in for this experiment.

A similar argument can be made for theorem 3 of the main text, but we do not need it since Theorem S1 is a refinement of theorem 3 whenever the influence curve is a step function, which is the case for MDRT2019.

3 Accuracy of estimates for M and d

Figure S2 shows the standard error of the estimates of M and d found in the data at the level of individual experimental items. We now describe how these standard errors are obtained.

Recall that for the V2019 and FV2021 datasets, we estimate d as $p_{ind}^B - p_{ind}^A = 1 - 2 \cdot p_{ind}^A$, where p_{ind}^A (p_{ind}^B) is the probability of choosing A (B) in the control condition. Therefore, $\text{se}(d) = 2 \cdot \text{se}(p_{ind}^A)$, where $\text{se}(X)$ denotes the standard error in the estimate of a random variable X. We estimate $\text{se}(p_{ind}^A)$ using the formula for the standard deviation of a binomial proportion $\sqrt{p_{ind}^A(1 - p_{ind}^A)/n}$, where n is the sample size (in this case, number of answers in the control condition) and in place of p_{ind}^A we plug in its point estimate (proportion of A answers).

For the MDRT2019 dataset, we have $d = p_{ind}^B - p_{ind}^A$, where p_{ind}^A (p_{ind}^B) now refers to the probability that members of party A (B) agree with the statement in the control condition. We obtain $\text{se}(p_{ind}^A)$ and $\text{se}(p_{ind}^B)$ as described above and set $\text{se}(d) = \sqrt{\text{se}(p_{ind}^A)^2 + \text{se}(p_{ind}^B)^2}$.

For M, in the cases of V2019 and FV2021 we have $M = p^+ - p^-$, where p^+ (p^-) is the probability estimate of choosing A when $0.5 < x < 0.5 + \epsilon$ ($0.5 - \epsilon < x < 0.5$), with ϵ being the bin size. Hence $\text{se}(M) = \sqrt{\text{se}(p^+)^2 + \text{se}(p^-)^2}$. The quantities $\text{se}(p^+)$ and $\text{se}(p^-)$ are obtained analogously to $\text{se}(p_{ind}^A)$ above. For the MDRT2019 dataset, we first apply this method

to estimate the standard errors of the marginal majority effects for the two parties separately ($\mathrm{se}(M_A)$ and $\mathrm{se}(M_B)$). Because $M = M_A + M_B$, we have $\mathrm{se}(M) = \sqrt{\mathrm{se}(M_A)^2 + \mathrm{se}(M_B)^2}$.

4 Different numbers of bins

To check the robustness of our results in the main text, we repeat the analysis with different numbers of bins (for the x-axis) in estimating the influence curves. Specifically, we reproduce Figs. 6, 8, and 9 of the main text, with the number of bins equal to $n = 8$, 12, and 14, as opposed to $n = 10$ in the main text. Note that Fig. 9 depends on the number of bins used, because the parameter M is calculated as the difference between the values of the influence curve in the two central bins. For example when $n = 10$ bins are used, the marginal majority effect is the difference between the choice probability for popularities $x \in (0.4, 0.5)$ and $x \in (0.5, 0.6)$. As we increase the number of bins, we restrict these intervals to be closer to 0.5. In all cases, the results are qualitatively similar to the ones in the main text (Figs. S3 to S7).

Furthermore, Fig. S8 shows how the estimation of the marginal majority effect M varies as a function of the bin size used. These estimates remain relatively robust, as long as they are based on sufficient data. Moreover, the lack of a clear downward trend shows that the marginal majority effect represents a "near-discontinuity", rather than a gradual increase near $x = 0.5$.

These results show robustness of the descriptive statistics reported in the main text.

5 Out-of-sample predictions

In the empirical section of the main text, data from all trials were used both to estimate the influence curves and to check whether lock-in occurred.[1] To see why this can be problematic, note that in principle we don't know whether the influence curve model is correct. It is conceivable that people do not pay attention to the proportions but instead to some other function of the counts, in a way that the process cannot be described via an influence curve.[2] For example, the proportions of people who choose each item need not converge, or they might not always converge to one of a small set of points (e.g., the stable equilibria of f). In that case, the proportion estimates for the equilibria would be merely properties of the available data, rather than estimates of an underlying ground truth. In particular, out-of-sample predictions would turn out to be inaccurate.

Here, we provide evidence that the model makes accurate out-of-sample predictions of the end-of-trial proportions and lock-in, which gives further support for the suitability of the model. Specifically, we repeat the analysis reported in the main text, but splitting the trials for each experimental item (question) into two sets, the first of which we use for estimating the influence curve and marginal majority effect (training set) and the second for assessing whether lock-in occurs (test set). Because V2019 included only two trials, we exclude it from the analysis.

5.1 Splitting the data into training and test sets

For MDRT2019 we use half of the trials (4/8) of each question for training and the rest for testing. For FV2021 the training set consists of half of the trials with 100 participants (5/10 or 8/15 trials, depending on the question) and all of the trials with 15 participants (20 or 30 trials, depending on the question), because the latter are too short to reveal the occurrence of lock-in. The rest of the trials with 100 participants form the test set.

In order to reduce any bias in the choice of how we split the trials in the figures that we include here, we have ordered the trials by their index in the original datasets that are available

[1] In V2019 a third trial contained external manipulations of the popularity and was used only for influence curve estimation. Also, in FV2021, trials of only 15 participants were used only for influence curve estimation.

[2] This is not relevant for MDRT2019, because users were not shown the counts of previous answers.

online and assigned the first half of the eligible trials to the training set. We also provide summary statistics from replications of the analyses with random splits.

5.2 Predicting lock-in-proneness from influence curves

Figures S9 and S10 show the influence curves and the predicted equilibria for MDRT2019 and FV2021, respectively, generated from the training set data, and the end-of-trial majorities for the test set trials.

In Fig. S9, there is good agreement between the predicted equilibria and observed end-of-trials proportions. Additionally, like in Fig. 6 of the main text, in all but one case the possibility of lock-in is predicted. Although only four trials for each question were used to assess the occurrence of lock-in, lock-in was observed in 16/19 cases where it was predicted. The one case where the influence curve is above the $y = 0$ line *on the left half of the graph* is due to very small sample size, only 7 answers from Democrats and Republicans together. For all other estimates there were at least 170 answers, and at least 70 from each group (Democrats and Republicans).

In Fig. S10, the right half of the graph is substantially affected by noise, due to the limited data available. Still, lock-in is observed (end-of-trial majority of option A) in 9 out of 13 cases (69%) in which it is predicted to be possible, while there are 2 out of 17 cases (12%) in which lock-in is observed without being predicted. With regards to the location of the end-of-trial proportion, the predictions (downcrossings) are reasonably accurate when option B has an end-of-trial majority (left half of the graph), but less so in the case of lock-in (right half of the graph), again presumably due to the limited amount of data.

Robustness check

The results are similar with different splits of the data into training and test sets. In 100 runs with random split,[3] we find that the influence curves of 27.8 out of 50 items on average entered the lock-in region (aggregate data for MDRT2019 and FV2021), and in 21.6 of those (77.7%) lock-in was observed. In contrast, in only 5.8/21.4 items on average (27.1%) lock-in was observed when the influence curves did not enter the lock-in region. (See main text for a discussion of the possible sources of error.) For 0.8 items on average there was no data for popularities $x > 0.5$. The confusion matrix is reported in Table S2. These results provide moderate support to the suitability of the theoretical model for describing the dynamics of popularity in these studies, as well as for the predictive validity of Theorem 1 in the main text.

5.3 Predicting lock-in-proneness from marginal majority effects

Figure S11 is a replication of Fig. 9 of the main text, with out-of-sample prediction, using the training data to estimate the marginal majority effect and the test data to decide whether lock-in is observed.[4] The split into training and test sets is described in Section 5.1. We see that the difference between marginal majority effect and quality difference is a good predictor of the possibility of lock-in. Specifically, lock-in is observed in 22/28 cases (79%) where it is predicted to be possible ($M > d$), and in 5/19 (26%) cases when $M < d$ (in which case the theory is inconclusive about its possibility).

[3] Sizes of test and training sets are as before. In FV2021 all trials of 15 participants are part of the training set.

[4] The difference in inherent appeal is estimated from the data of the control experiment, similarly to Fig. 9 of the main text.

Robustness check

The results are similar when the split between training and test sets is random. Specifically, for the two experiments together, in 100 runs with random split, the condition $M > d$ was satisfied for 28.5 items on average (lock-in predicted), and for 23.7 of those on average lock-in was observed (83%). In contrast, lock-in was observed on average for only 3.7/18.1 (20.4%) of the items for which $M < d$ (and for which there is no theoretical prediction). For 3.4 items on average, there was no data for estimating M. The confusion matrix is reported in Table S3.

These predictions are more accurate than those based on the influence curve entering the lock-in region (Section 5.2), despite using less data, and they provide moderate support for the predictive validity of Theorem 2 in the main text.

5.4 Predicting the frequency of lock-in

We also repeated the analysis regarding the number of lock-in occurrences, for out-of-sample predictions. We split the trials into training and test sets as described in Section 5.1. For each item, we estimated M and d from the training set and used Eq. (2) from the main text to find lower bounds for the lock-in probability. We then used those estimates of the lock-in probabilities to find the minimum number of lock-in occurrences we should expect to observe in the test set trials. We find that with 95% confidence, lock-in must occur in at least 14 out of 135 total trials for 28 items that satisfy the condition $M > d$. The empirical number of lock-in occurrences in those trials is 40, consistent with the prediction. For comparison, in 117 trials for 26 items that did not satisfy the condition $M > d$,[5] lock-in occurs only 6 times. The results are similar when we use different, random splits of the trials into training and test sets. This provides moderate support for the predictive validity of Theorem 3 in the main text.

[5]For three items, there was no data in the training set to estimate M.

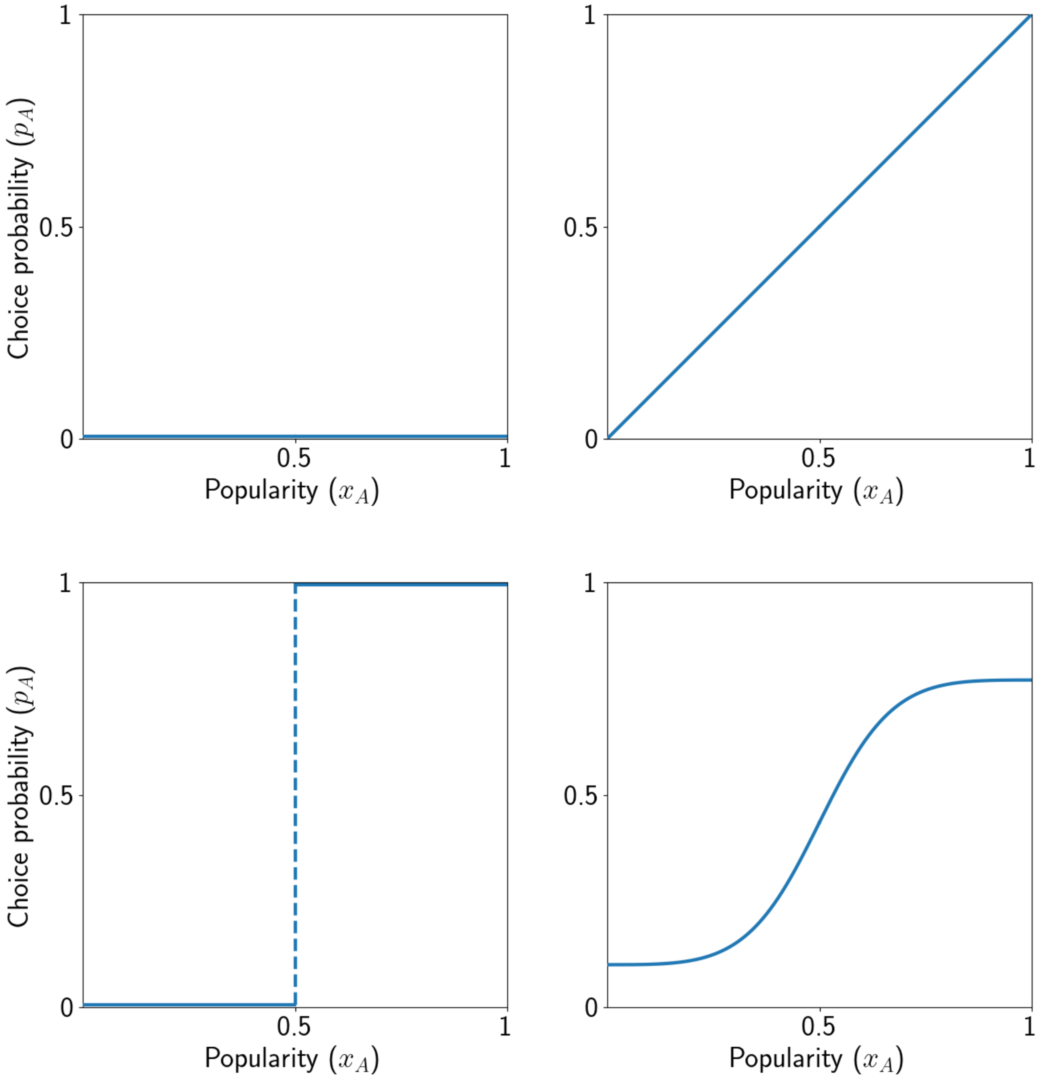

Figure S1: Possible individual-level influence curves. Top left: Influence curve describing an individual who never chooses option A, no matter how many others have chosen it. **Top right:** An individual who chooses each option with probability equal to its popularity. **Bottom left:** An individual following the majority heuristic. **Bottom right:** An individual whose probability of choosing A is an arbitrary, non-linear function of its popularity.

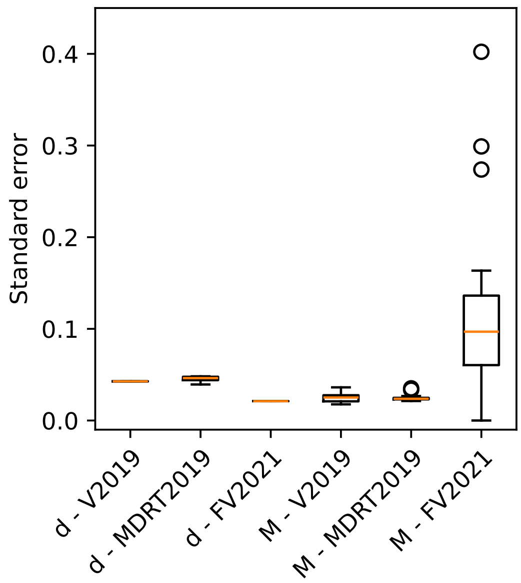

Figure S2: Boxplots of the standard error across items for the estimates of d and M in each dataset.

V2019 - Varying number of bins

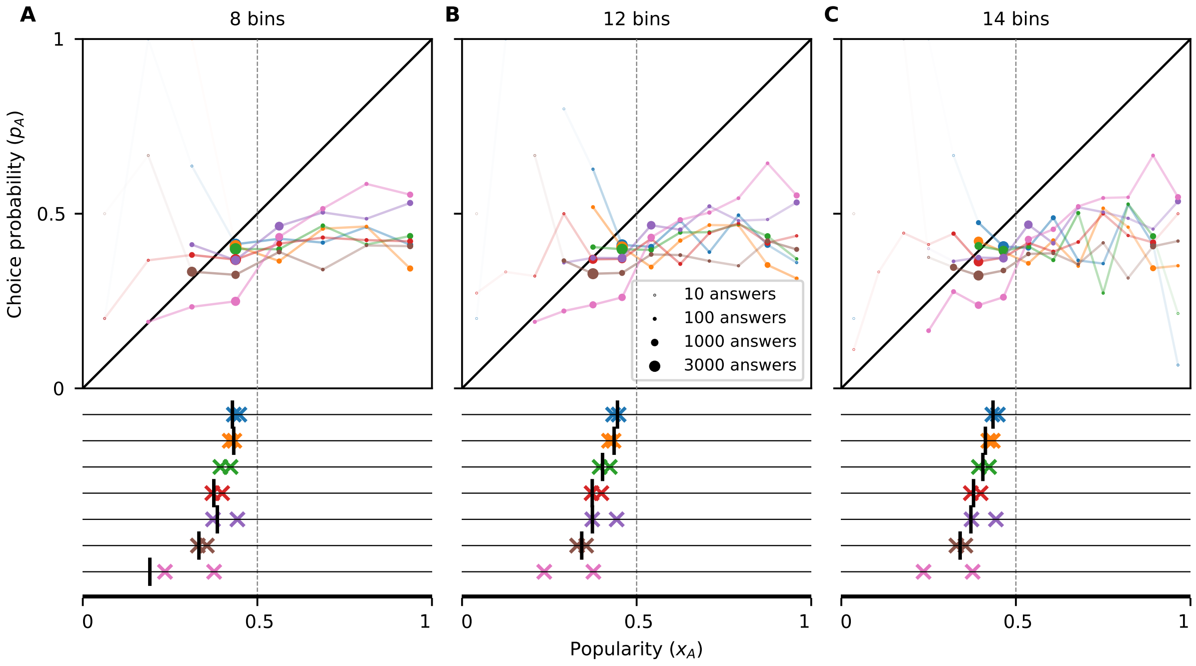

Figure S3: Replication of Fig. 6 of the main text, with different numbers of bins. Color coding of the items is the same as in Fig. 6.

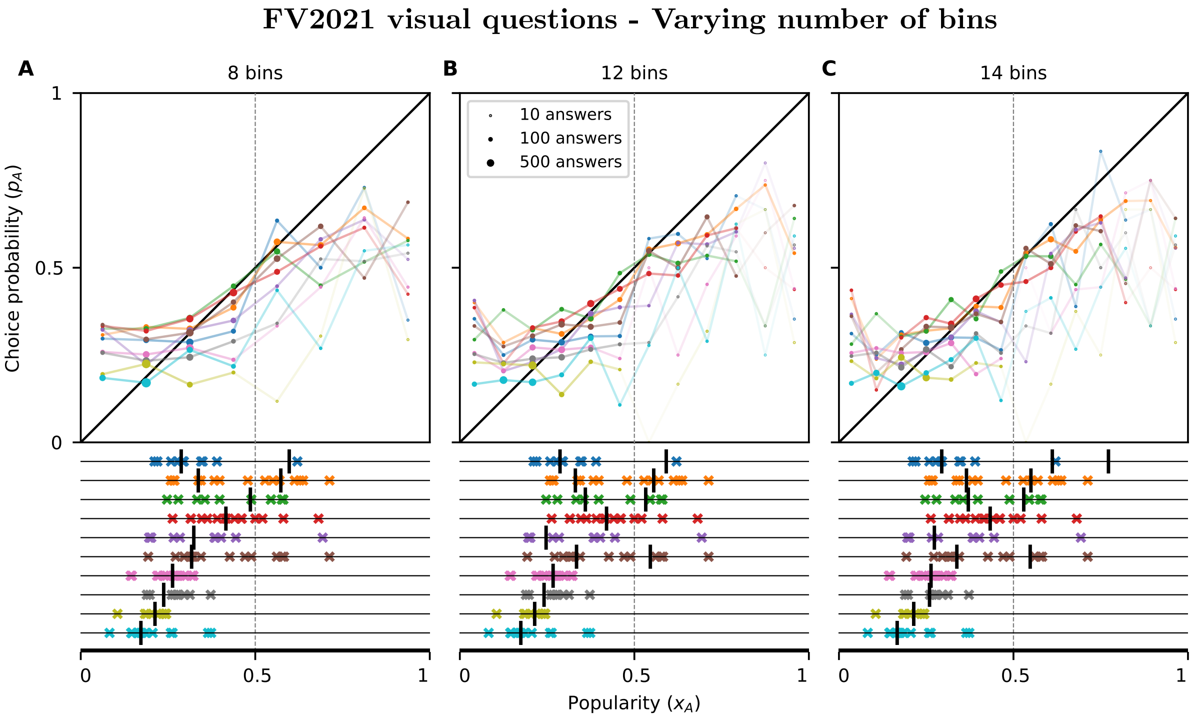

Figure S4: Replication of Fig. 8A of the main text, with different numbers of bins. Color coding of the items is the same as in Fig. 8A.

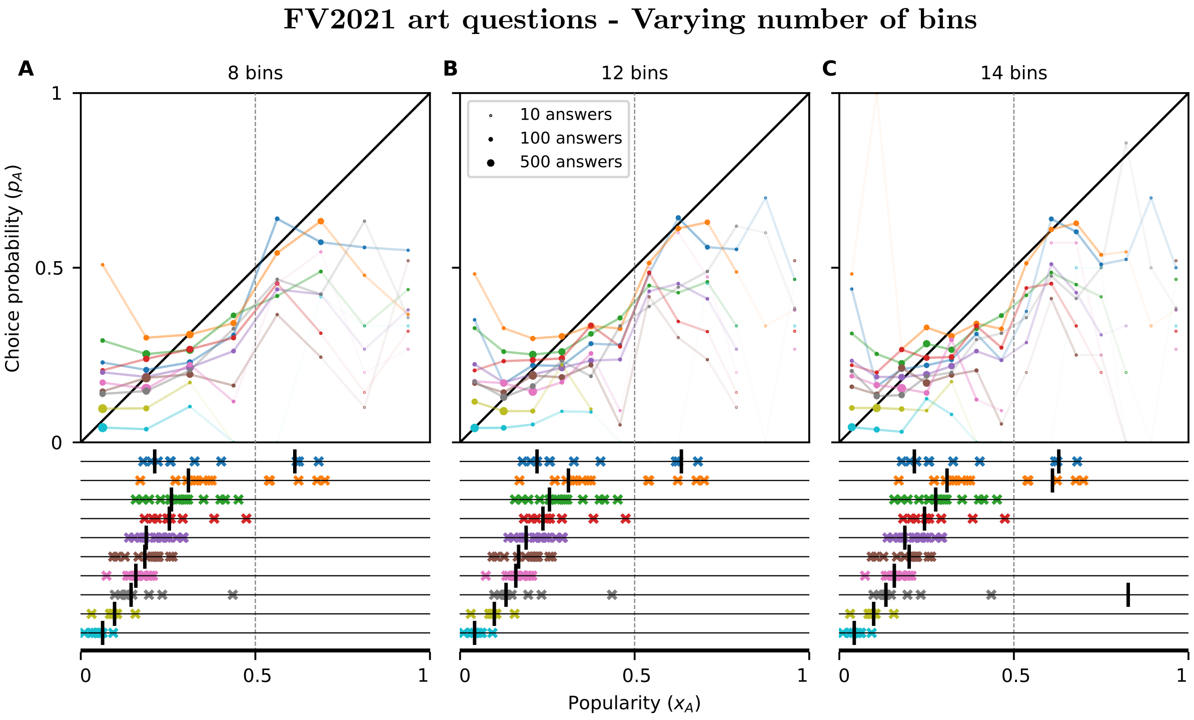

Figure S5: Replication of Fig. 8B of the main text, with different numbers of bins. Color coding of the items is the same as in Fig. 8B.

FV2021 geometry questions - Varying number of bins

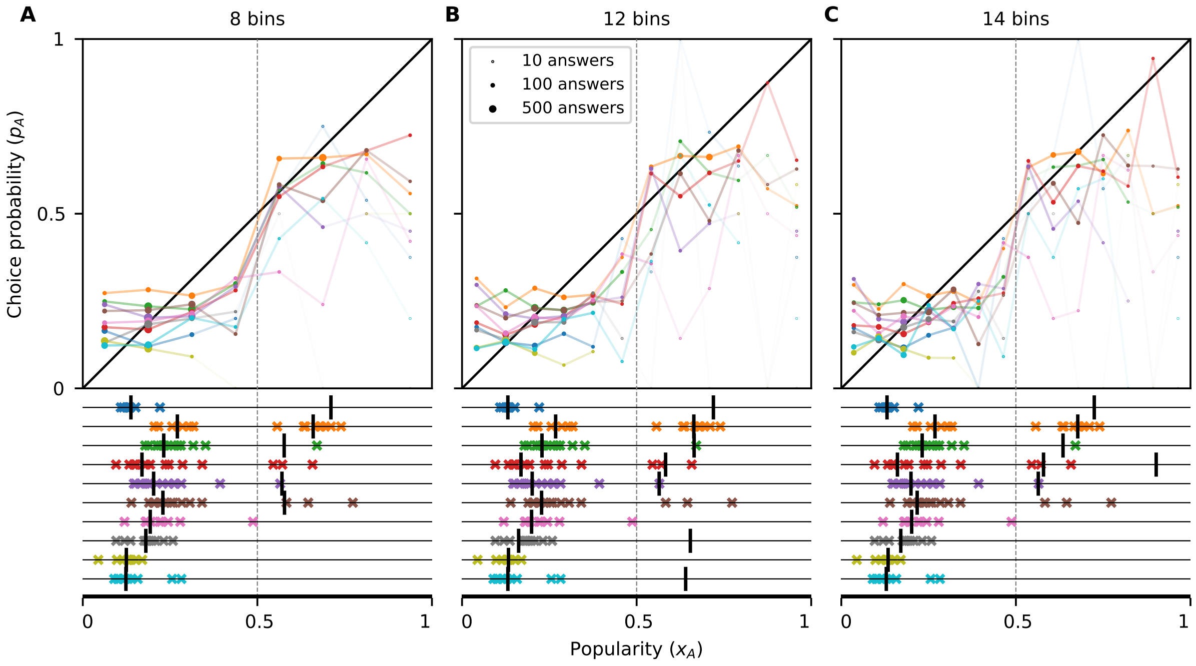

Figure S6: Replication of Fig. 8C of the main text, with different numbers of bins. Color coding of the items is the same as in Fig. 8C.

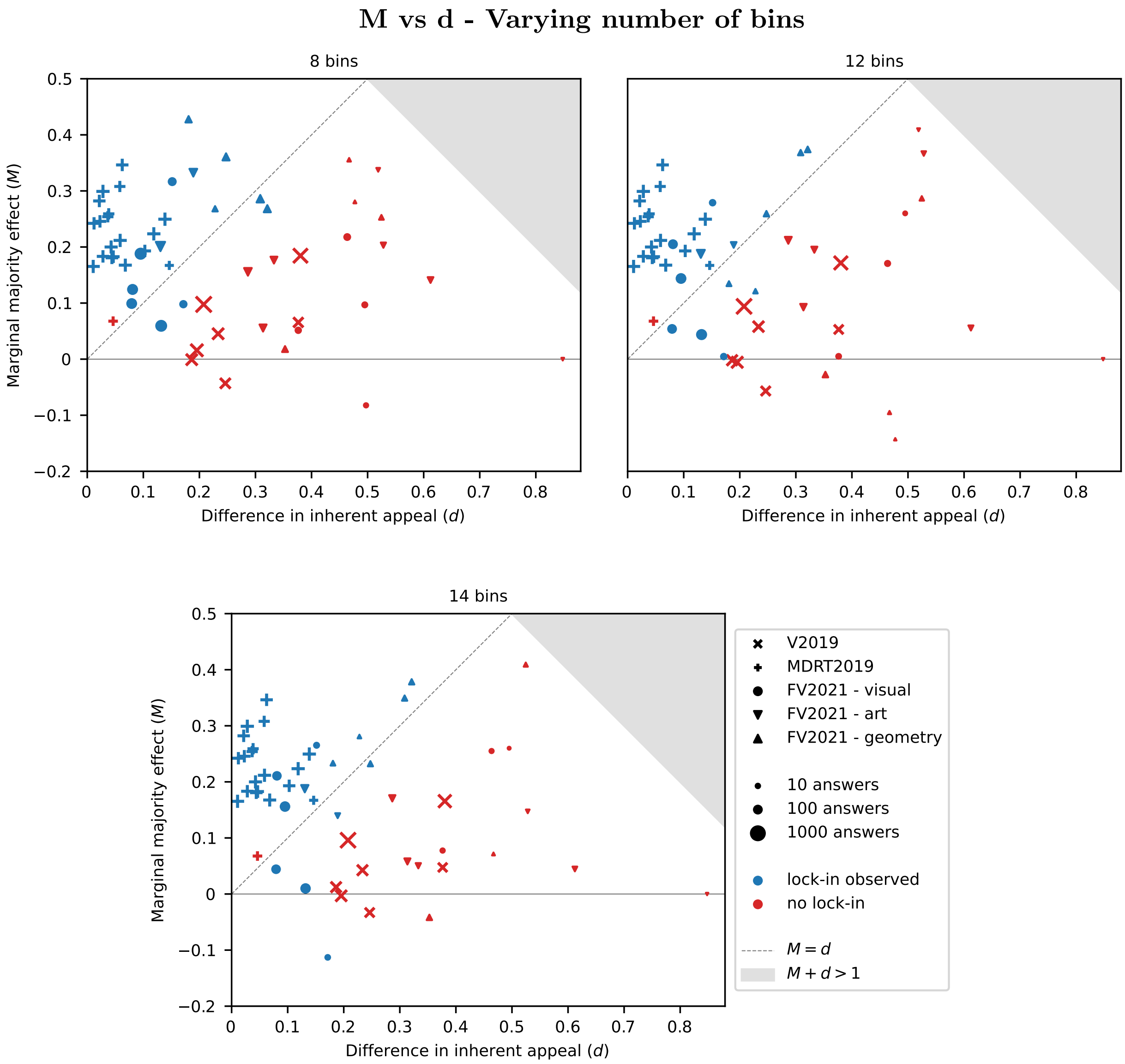

Figure S7: Replication of Fig. 9 of the main text, with different numbers of bins.

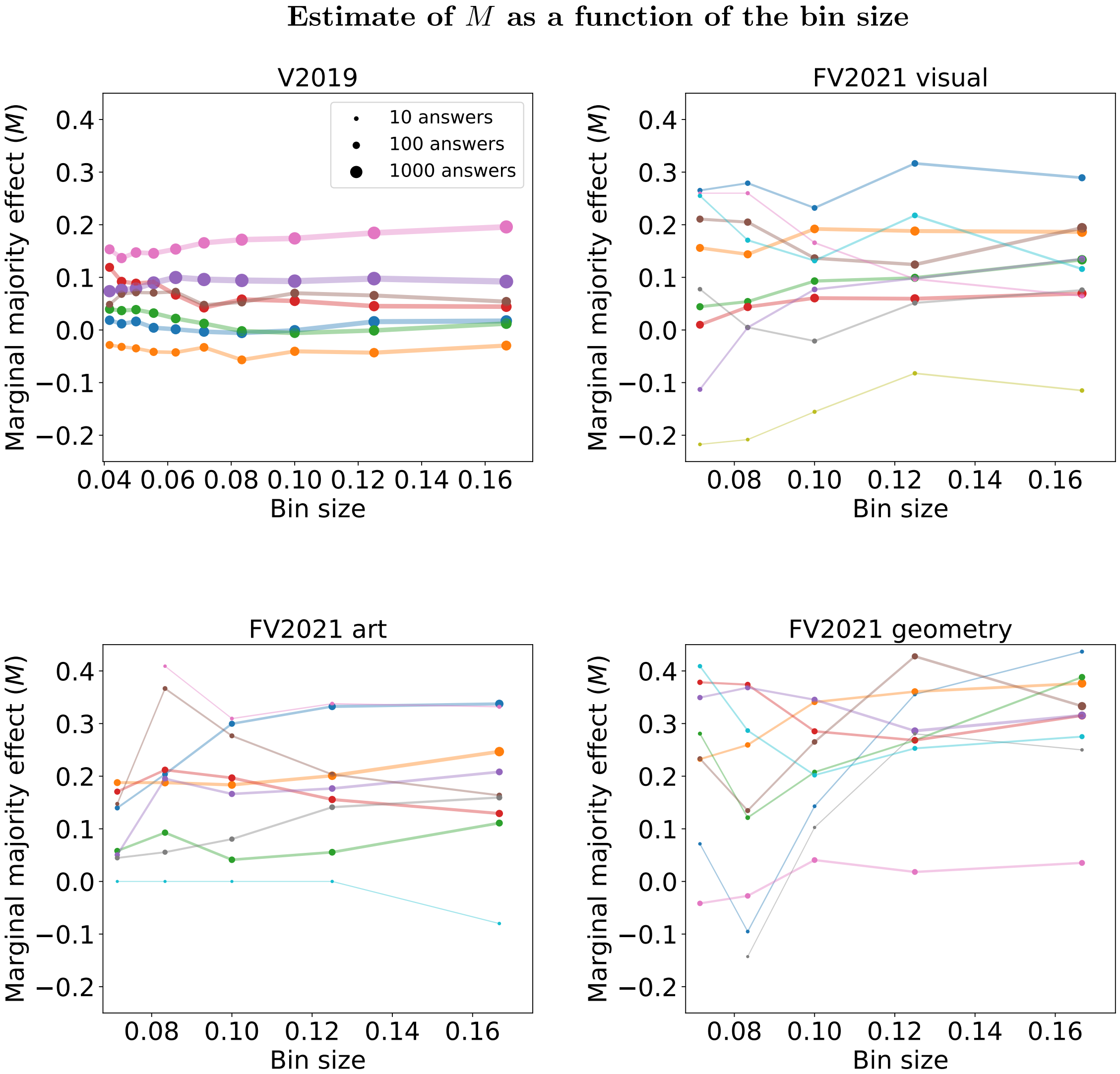

Figure S8: Marginal majority effect as a function of the bin size. For the V2019 and FV2021 datasets, the marginal majority effect is estimated as the difference between the choice probability in the two central bins $(0.5 - l, 0.5)$ and $(0.5, 0.5 + l)$, where l is the bin size. Color coding of the items is the same as in Figs. 6 and 8 of the main text.

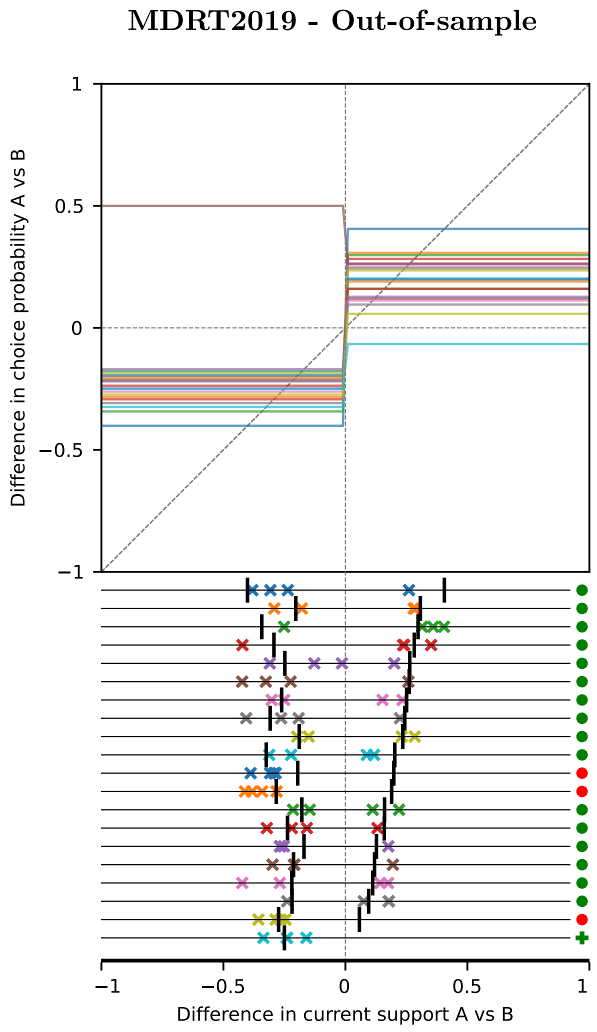

Figure S9: Out-of-sample prediction of lock-in-proneness for the MDRT2019 dataset from influence curves. This is a replication of Fig. 7 of the main text, but using a subset of the trials of each question for estimating the influence curve and the rest of the trials for calculating end-of-trial proportions. The marks at the bottom right of the graph indicate for which of the questions the predictions are accurate. **Circles/crosses:** Lock-in predicted/not predicted based on influence curve entering or not the lock-in region. **Green/red color:** Prediction accurate/not accurate. Color coding of the items is the same as in Fig. 7.

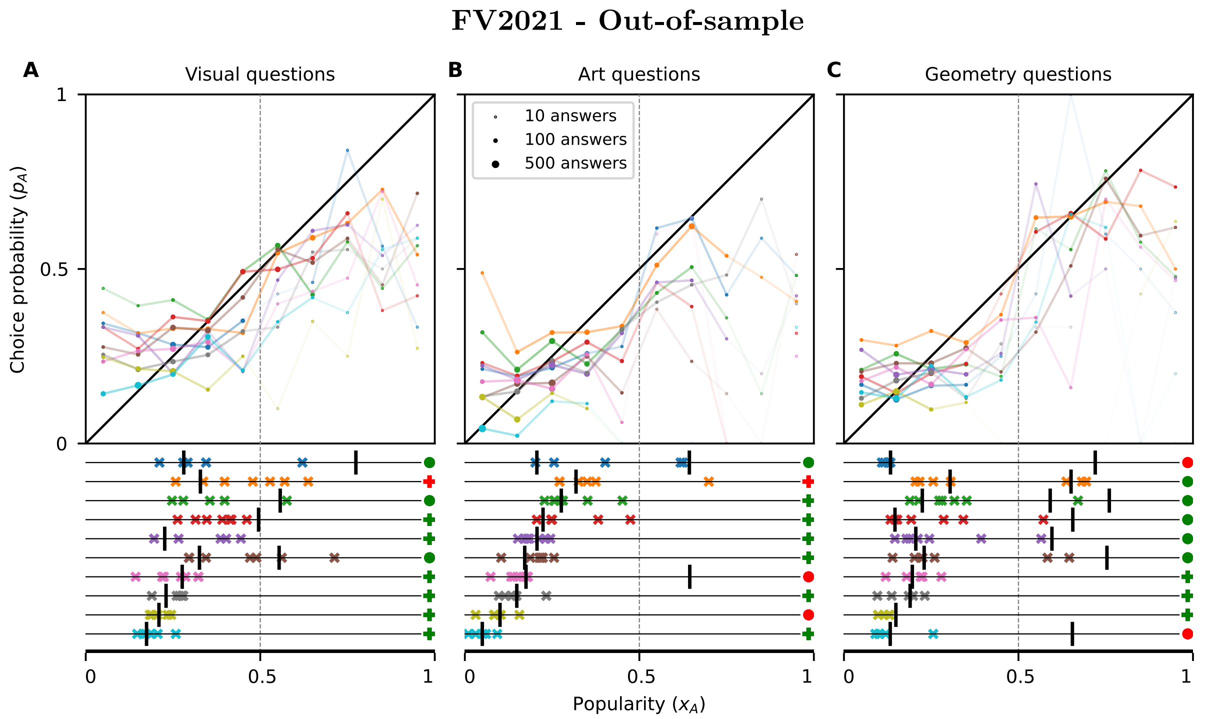

Figure S10: Out-of-sample prediction of lock-in-proneness for the FV2021 dataset from influence curves. Similar to Fig. S9, for the FV2021 dataset. The corresponding figure in the main text is Fig. 8. Color coding of the items is the same as in Fig. 8.

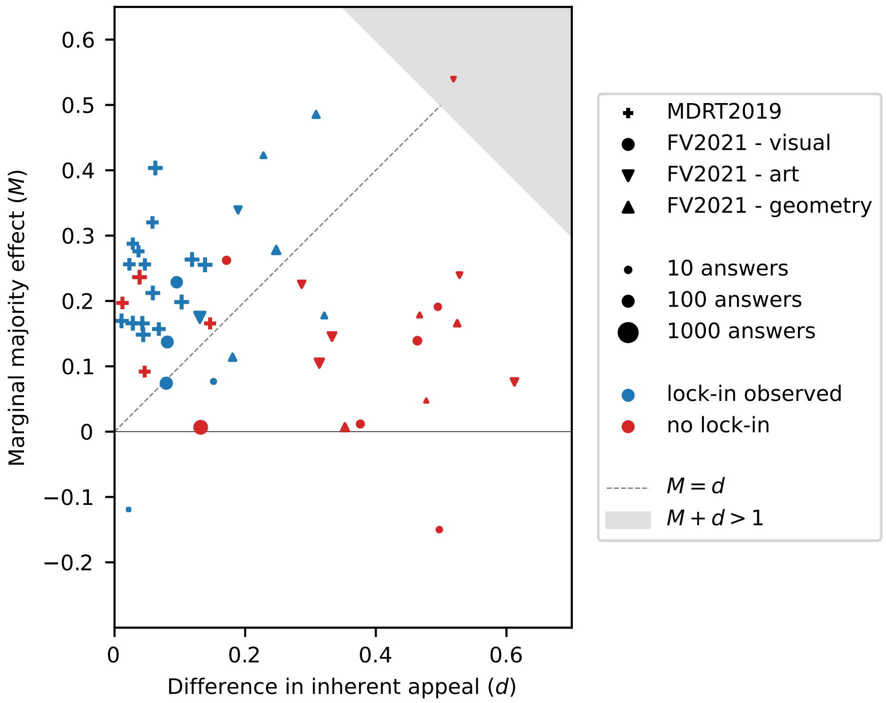

Figure S11: Out-of-sample prediction of lock-in-proneness from marginal majority effects and differences in inherent appeal. This is a replication of Fig. 9 of the main text, but using a subset of the trials of each question for estimating the marginal majority effect and the rest of the trials for assessing the occurrence of lock-in. Note that although the gray-shaded region ($M + d > 1$) is impossible in theory, it can occur empirically because we are estimating d and M from different sets of data (d is estimated from the control condition of the experiment, without social influence).

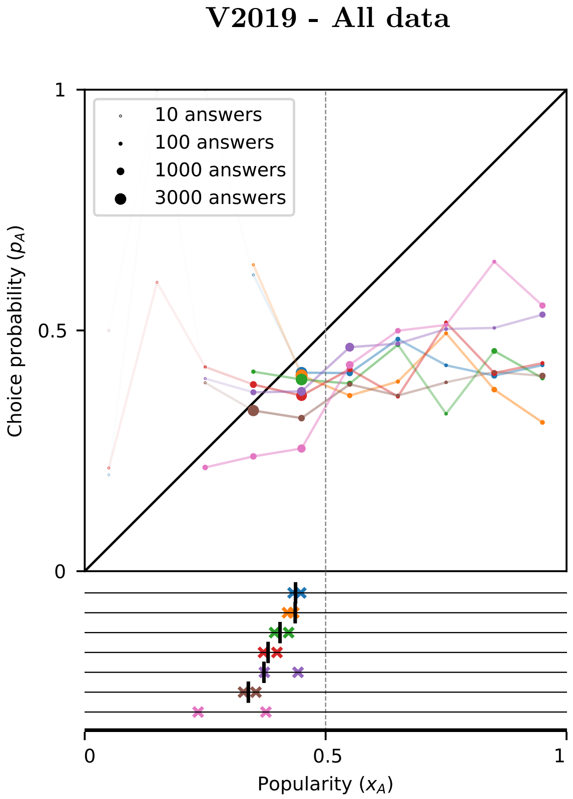

Figure S12: Replication of Fig. 6 of the main text, without removing data at $x = 0.5$. In Fig. 6 of the main text, points that fell exactly on $x = 0.5$ were removed from the graph, in order for the marginal majority effect to be visible (see Materials and Methods in the main text). Here we replicate that figure without removing those points, but instead treating them the same way we treat other points that fall on bin endpoints, i.e., by placing them with weight 0.5 on each of the adjacent bins. Color coding of the items is the same as in Fig. 6.

FV2021 - All data

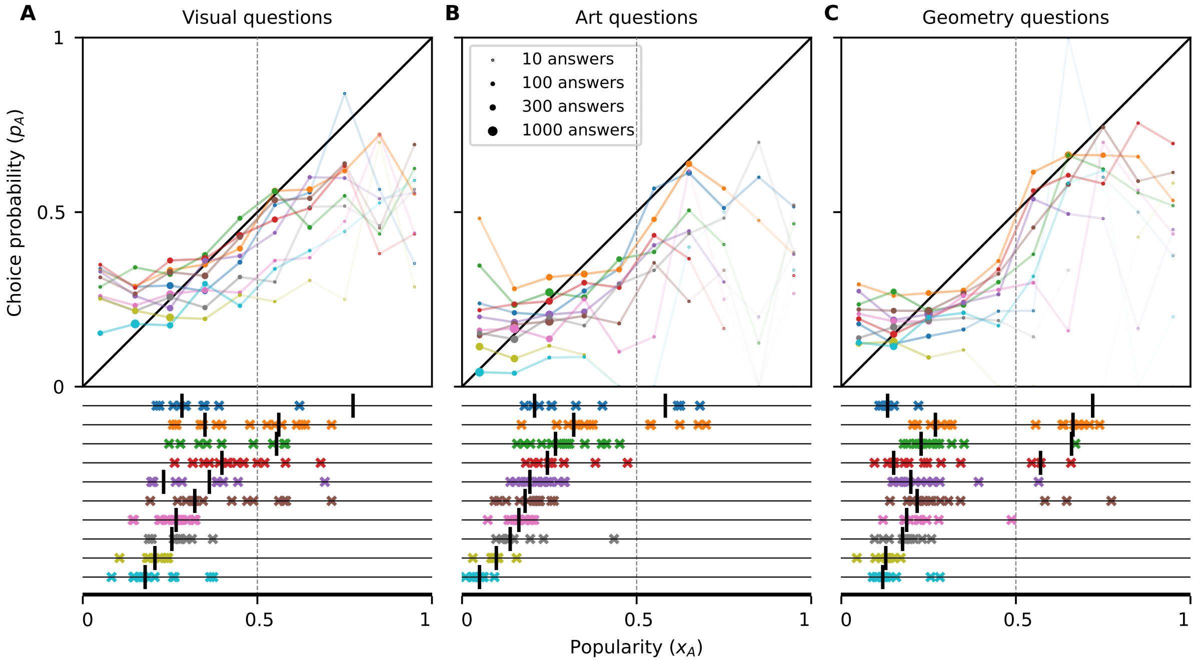

Figure S13: Replication of Fig. 8 of the main text, without removing data with $x = 0.5$. Similar to Fig. S12, for the FV2021 dataset. Color coding of the items is the same as in Fig. 8.

Table S1: Summary of lock-in probabilities, theoretical confidence intervals for number of lock-in occurrences obtained from Eq. (S1), and empirical numbers of lock-in occurrences for the MDRT2019 dataset. The order of the questions is the same as in Fig. 7 of the main text.

Question	M	d	p_L (per trial)	Num. trials	95% CI	Observed
4	0.346	0.062	0.402	8	[1, 6]	3
11	0.308	0.058	0.399	8	[1, 6]	6
16	0.250	0.139	0.213	8	[0, 4]	4
15	0.299	0.028	0.450	8	[1, 6]	4
19	0.282	0.022	0.459	8	[1, 6]	5
17	0.212	0.059	0.357	8	[0, 6]	4
13	0.246	0.023	0.452	8	[1, 6]	5
6	0.254	0.037	0.424	8	[1, 6]	2
5	0.242	0.012	0.474	8	[1, 7]	3
9	0.259	0.038	0.422	8	[1, 6]	2
18	0.165	0.011	0.467	8	[1, 6]	4
7	0.223	0.119	0.227	8	[0, 4]	4
20	0.193	0.103	0.228	8	[0, 4]	3
2	0.183	0.028	0.421	8	[1, 6]	3
12	0.200	0.043	0.390	8	[1, 6]	4
8	0.181	0.044	0.375	8	[1, 6]	3
1	0.168	0.068	0.293	8	[0, 5]	4
10	0.183	0.046	0.370	8	[1, 6]	2
3	0.167	0.146	0.06	8	[0, 2]	1
14	0.068	0.046	0.159	8	[0, 4]	0
**All**				160	[45, 68]	66

Table S2: Confusion matrix for the lock-in prediction based on whether the influence curve enters the lock-in region. The numbers are averages over 100 runs with random splits of the trials into training and test sets, for MDRT2019 and FV2021 together.

Lock-in	Predicted	Not predicted	**Total**
Observed	21.6	6.2	**27.8**
Not Observed	5.8	15.6	**21.4**
**Total**	**27.4**	**21.8**	**49.2**

Table S3: Confusion matrices for the lock-in prediction based on the condition $M > d$. The numbers are averages over 100 runs with random splits of the trials into training and test sets, for MDRT2019 and FV2021 together. Note that when $M \leq d$, there is no theoretical prediction, either for presence or absence of lock-in.

Lock-in	Predicted	No prediction	**Total**
Observed	23.7	3.7	**27.4**
Not Observed	4.8	14.4	**19.2**
**Total**	**28.5**	**18.1**	**46.6**

Table S4: Logistic Regression for lock-in-proneness.

	$Y \sim 1 + d$			$Y \sim (M - d)$		
Variable	β (95% CI)	SE	z	β (95% CI)	SE	z
Intercept	4.112***	1.053	3.907	—	—	—
x_1	−17.330***	4.560	−3.800	23.458***	6.968	3.367
	$(-26.267, -8.392)$			$(9.801, 37.114)$		
Model Fit Statistics						
Log-likelihood	−16.549			−10.545		
AIC	37.1			23.09		
BIC	41.113			25.096		
McFadden's R^2	0.557			0.718		
N observations	55			55		

β = logistic regression coefficient; SE = standard error; z = z-statistic; CI = confidence interval.

$^{***}p < 0.001$. All coefficients are significant at the 0.001 level.

Model 1: $\text{logit}(p) = 4.112 - 17.330 \times x_1$; Model 2: $\text{logit}(p) = 23.458 \times x_1$ (no intercept).

Model 2 demonstrates superior fit across all criteria (LL, AIC, BIC, R^2).

Table S5: Nadaraya-Watson Kernel Regression for lock-in-proneness.

	Model 1 (d)	Model 2 ($M - d$)
Model Fit Statistics		
Log-likelihood	-16.747	-6.74
R^2	0.62	0.864
N observations	55	55

Each model has a single regressor (d or $M - d$). A Gaussian kernel is used, whose standard deviation is fitted to minimize the leave-one-out cross-validation mean squared error.
Model 2 demonstrates superior fit with respect to both log-likelihood and R^2 metrics.

Table S6: Question ids in the original experiments, listed by the order they appear in Figs. 6-8 of the main text and all corresponding supplementary figures.

V2019	MDRT2019	FV2021 Visual	FV2021 Art	FV2021 Geometry
Wallpaper	4	6	102	204
Modern art	11	9	109	203
Music	16	10	101	207
Houseware	15	5	108	209
Elections	19	2	103	201
Visual test	17	3	107	205
Green ideas	13	1	105	208
	6	8	110	206
	5	4	104	210
	9	7	106	202
	18			
	7			
	20			
	2			
	12			
	8			
	1			
	10			
	3			
	14			
